# Selective influence of Sox2 on POU transcription factor binding in embryonic and neural stem cells

**DOI:** 10.15252/embr.201540467

**Published:** 2015-09-02

**Authors:** Tapan Kumar Mistri, Arun George Devasia, Lee Thean Chu, Wei Ping Ng, Florian Halbritter, Douglas Colby, Ben Martynoga, Simon R Tomlinson, Ian Chambers, Paul Robson, Thorsten Wohland

**Affiliations:** 1 Department of Chemistry, National University of SingaporeSingapore, Singapore; 2 Developmental Cellomics Laboratory, Genome Institute of SingaporeSingapore, Singapore; 3 MRC Centre for Regenerative Medicine, Institute for Stem Cell Research, School of Biological Sciences, University of EdinburghEdinburgh, UK; 4 Division of Molecular Neurobiology, MRC-National Institute for Medical ResearchMill Hill, London, UK; 5 Department of Biological Sciences, National University of SingaporeSingapore, Singapore; 6 The Jackson Laboratory for Genomic Medicine, FarmingtonCT, USA; 7 Centre for Bioimaging Sciences, National University of SingaporeSingapore, Singapore

**Keywords:** fluorescence correlation spectroscopy, fluorescent protein-based electrophoretic mobility shift assay, MORE, PORE, Sox/Oct motif

## Abstract

Embryonic stem cell (ESC) identity is orchestrated by co-operativity between the transcription factors (TFs) Sox2 and the class V POU-TF Oct4 at composite Sox/Oct motifs. Neural stem cells (NSCs) lack Oct4 but express Sox2 and class III POU-TFs Oct6, Brn1 and Brn2. This raises the question of how Sox2 interacts with POU-TFs to transcriptionally specify ESCs versus NSCs. Here, we show that Oct4 alone binds the Sox/Oct motif and the octamer-containing palindromic MORE equally well. Sox2 binding selectively increases the affinity of Oct4 for the Sox/Oct motif. In contrast, Oct6 binds preferentially to MORE and is unaffected by Sox2. ChIP-Seq in NSCs shows the MORE to be the most enriched motif for class III POU-TFs, including MORE subtypes, and that the Sox/Oct motif is not enriched. These results suggest that in NSCs, co-operativity between Sox2 and class III POU-TFs may not occur and that POU-TF-driven transcription uses predominantly the MORE *cis* architecture. Thus, distinct interactions between Sox2 and POU-TF subclasses distinguish pluripotent ESCs from multipotent NSCs, providing molecular insight into how Oct4 alone can convert NSCs to pluripotency.

## Introduction

Reprogramming of somatic cells to pluripotency is a landmark discovery in stem cell biology, fuelling novel regenerative medicine applications. Forced expression of four transcription factors (TFs) expressed in ESCs (Oct4, Sox2, c-Myc and Klf4) can reprogramme mouse embryonic fibroblasts to pluripotency 1. NSCs express Sox2, c-Myc and Klf4, but not Oct4 2. Consistent with this, NSCs can be driven to pluripotency by ectopic expression of Oct4 alone 3.

Oct4 is a TF of the highly conserved POU (Pit-Oct-Unc) domain family that includes general, developmental and tissue-specific regulators of many cell types 4-6. POU family TFs contain a common POU DNA-binding domain of approximately 150–160 amino acids consisting of an N-terminal POU-specific (POU_S_) subdomain of 75 amino acids and a C-terminal POU homeodomain (POU_HD_) of 60 amino acids connected by a flexible linker ranging in length from 15 to 56 amino acid residues. The bipartite modular nature of this DNA-binding domain enables the two subdomains to work separately in DNA recognition, transcriptional activity or functional interaction with other cofactors involved in gene regulation 7, 8.

POU-TFs control gene expression by binding to target sequences either by homodimerisation or by heterodimerisation with other TFs 9, 10. Several studies have shown that Oct4, encoded by the *Pou5f1* gene 11, 12, 13, 14, can heterodimerise with Sox2 through the Sox2 high mobility group (HMG) DNA-binding domain in a DNA-dependent manner 15, 16, 17. This heterodimer binds to Sox/Oct motifs present in *cis* at many ESC-expressed genes to regulate their expression including *Nanog, Fgf4, Utf1* and *Fbx15* as well as the *Sox2* and *Pou5f1* genes themselves 16, 18, 19, 20, 21, 22. The extent to which the Sox/Oct co-motif is bound by Sox2 and Oct4 became apparent following application of genome-wide chromatin immunoprecipitation (ChIP) methods to ESCs. The top motif identified using *de novo* motif discovery algorithms applied independently to Sox2 and Oct4 ChIP data sets from both human 23 and mouse 24, 25 ESCs was a Sox/Oct motif (CATTGTTATGCAAAT), which is a simple composite between a Sox2 binding site (CATTGTT) and an octameric Oct4 binding site (ATGCAAAT).

Prior to the emergence of genome-wide binding data, *in vitro* binding studies of Oct4 on naked DNA showed that Oct4 could also form a homodimer on a palindromic Oct factor recognition element (PORE; ATTTGAAATGCAAAT) 9. Moreover, different POU factors can homodimerise on this pseudo-palindromic PORE or on a second true palindrome, the MORE (More palindromic Oct factor recognition element) ATGCATATGCAT 26, 27, 28. Despite this, genome-wide ChIP data sets have provided little evidence that either the PORE or the MORE plays a significant role in the recruitment of Oct4 to binding sites in the ESC genome.

These findings raise the question of what regulates the choice between heterodimer and homodimer formation, and therefore genome localisation, by POU factors. It has previously been shown that altering the Sox factor partnering from Sox2 in ESCs to Sox17 in primitive endoderm-like cells switches the Oct4 binding locations and that this effect is mediated through differing Sox/Oct motifs 29. However, whether Sox2 affects the behaviour of Oct4 homologues in other cellular contexts is unknown. NSCs provide an intriguing model to explore this because they express Sox2 and the class III POU-TFs Oct6/*Pou3f1*, Brn1/*Pou3f3* and Brn2/*Pou3f2* 3, 30. Moreover, recent data suggest that Sox2 is extensively co-localised with Brn2 in neural progenitor cell chromatin 30, reminiscent of the co-localisation of Oct4 and Sox2 in ESCs. However, NSCs can be reprogrammed to a pluripotent identity by the expression of Oct4 alone 3, 31, suggesting that there may be a fundamental distinction in the way that the various POU-TFs respond to Sox2 and that these differences may be important for establishing and maintaining cell identity.

To examine this, the binding affinities of Oct4 and the representative NSC-expressed POU-TF, Oct6, for DNA elements containing the Sox/Oct, PORE and MORE motifs in the presence and in the absence of Sox2 were compared. Data from fluorescent protein-based electrophoretic mobility shift assays (FP-EMSA) and ChIP-Seq suggest the hypothesis that on DNA, the class V POU-TF Oct4 prefers to bind co-operatively with Sox2, while class III POU-TFs Oct6, Brn1 and Brn2 prefer homodimerisation. This illustrates how enforced expression of Oct4 in NSCs can change the pattern of Sox2 interactions to initiate novel target gene expression through Sox/Oct motifs.

## Results

### Oct4 binds to the Sox/Oct motif synergistically with Sox2 but forms homodimers on PORE

Oct4 and Sox2 have previously been demonstrated to bind synergistically to the Sox/Oct motif 16, 22, 32, 33. However, quantitative assessment of the synergistic affinity of DNA binding by full-length Oct4 and Sox2 protein has not been obtained. We used full-length Oct4 and Sox2, tagged at their N-termini with GFP and mCherry, respectively, to enable FP-EMSA, a modification of the EMSA technique in which the contribution of a protein to a protein–DNA complex can be visualised through the use of fluorescence protein tags 17, 34. First, however, the biological activity of the FP fusions was compared to wild-type Oct4 and Sox2 by determining the ability of proteins to prevent ESC differentiation upon silencing or deletion of *Oct4* or *Sox2* alleles. This showed that GFP-Oct4 and mCherry-Sox2 had comparable biological activities to Oct4 and Sox2, respectively (Appendix Fig S1). The utility of the FP-EMSA approach was then determined by comparing the detection of fluorescently tagged or untagged DNA with the detection of fluorescently tagged proteins. The same monomer and dimer complexes could be detected by retardation of either fluorescent protein mobility or conventionally labelled DNA, confirming that protein–DNA complex formation could be assessed by FP-EMSA (FigEV1A). Similar results were obtained using a mCherry–Oct4 fusion, indicating that dimer formation in FP-EMSA occurs independently of the fluorescent protein identity (FigEV1B).

**Figure:**
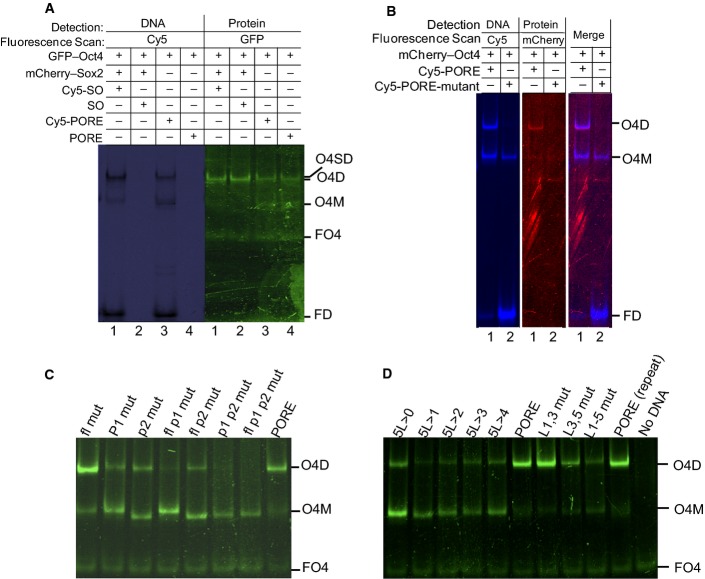
Validating FP-EMSA Homodimer formation was tested using DNA labelled with Cy5 or unlabelled as indicated. The presence of a Cy5 tag does not affect protein binding as dimerisation in lanes 2 and 4 is comparable with lanes 1 and 3.

Homodimerisation is independent of the fluorescent protein tag. mCherry–Oct4 was tested instead of GFP-Oct4 with PORE-wt (lane 1) and PORE-mt (lane 2).

Mutations inside the PORE motif affect homodimer formation, whereas mutations outside the PORE motif do not. The wild-type PORE is indicated; fl, P1 and P2 indicate oligonucleotides with mutations in the flanking sequence, palindromic repeat 1 and repeat 2, respectively.

Effects of mutations of base pairs in the non-palindromic linker of the PORE motif (AAATG). 5L > 4-0 indicates sequential reduction in the size of the linker; other point mutation oligonucleotides are indicated. Data information: O4D: GFP-Oct4 homodimer complex, O4SD: GFP-Oct4 and mCherry-Sox2 heterodimer complex, O4M: GFP-Oct4 monomer complex, FO4: free GFP-Oct4, and FD: free DNA. All oligonucleotide sequences are in Appendix Table S1. *n *=* *3. Source data are available online for this figure.

**Figure 1:**
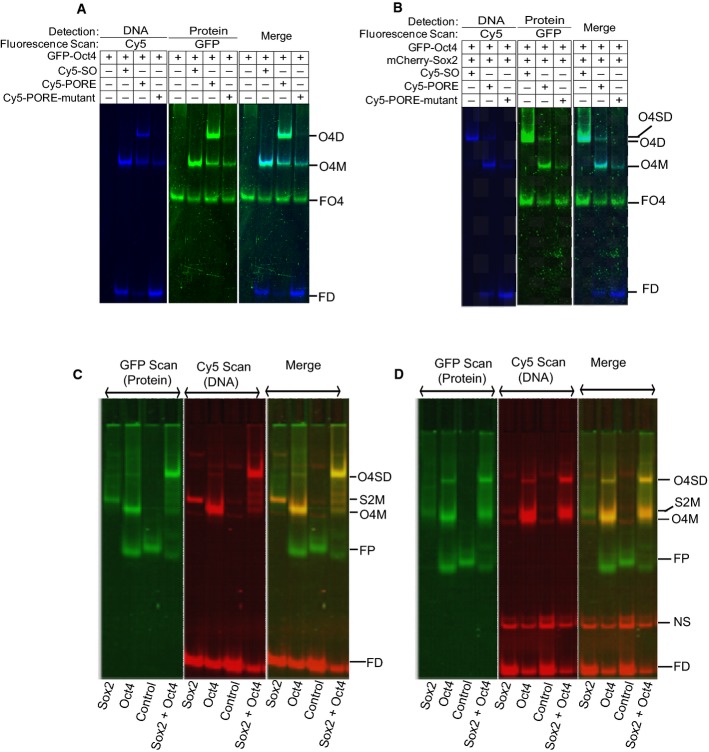
Oct4 binding to the Sox/Oct motif and specificity for the PORE A, B EMSA (DNA detection) and FP-EMSA (protein detection) of GFP–Oct4 interaction with the *Nanog* Sox/Oct motif or with PORE motifs, in the absence (A) or presence (B) of mCherry-Sox2.

GFP-Oct4 forms a monomer on the *Nanog* Sox/Oct motif and a monomer and a homodimer on the PORE motif (Fig1A). Dimer but not monomer formation on the PORE is abolished by mutation of all five bases in one half of the palindromic PORE sequence, leaving a single retarded DNA:Oct4 species with the same mobility as formed by binding of Oct4 to the *Nanog* Sox/Oct motif (Fig1A). Interestingly, mutation of only the central 3 bp of either 5 bp palindromic component of the PORE motif reduced but did not abolish homodimer formation, while simultaneous mutation of the central 3 bp of both palindromic components eliminated homodimer formation (Fig EV1C). In contrast, mutations outside of the motif have no effect. The importance of the spacer (AAATG) between the POU domain binding sites (ATTTGaaatgCAAAT) was examined by substitution and deletion mutagenesis. Reducing spacer size decreases dimer formation but weak dimerisation can occur without a linker. Moreover, while some substitutions within the spacer are neutral, others reduce dimer formation, indicating that the spacer is an integral part of the PORE (FigEV1D). When complex formation by Oct4 was monitored in the presence of Sox2, binding of Oct4 to the *Nanog* Sox/Oct motif was strongly shifted from a weak monomeric band to a robust dimer, whereas binding to the PORE motif was unaffected by Sox2 (Fig1B). Together, these results indicate that the type of dimer formed by Oct4 on DNA is determined by the specific DNA motif and that the presence of Sox2 causes preferential binding of Oct4 to the *Nanog* Sox/Oct motif as a heterodimer.

C, D FP-EMSA of binding of Oct4 and Sox2 to the Nanog Sox/Oct motif (C) or to a Sox/Oct motif with a 3-bp insertion (D) between the Oct and Sox components of the Sox/Oct motif. In the presence of Oct4 and Sox2, the bands shift completely from the positions of monomers to the positions of heterodimers in (C), indicating synergistic binding, but not in (D). Data information: Control: a non-binding GFP-TF fusion protein, O4D: GFP-Oct4 homodimer complex, O4SD: GFP-Oct4/GFP-Sox2 dimer complex on DNA, S2M: GFP-Sox2 monomer on DNA motif, O4M: GFP-Oct4 monomer on DNA motif, FP: free protein, NS: non-specific binding, and FD: free Cy5-tagged DNA motif. All oligonucleotide sequences are listed in Appendix Table S1. *n *=* *3 for (A, B), 2 for (C, D). Source data are available online for this figure.

Visual comparison of the binding of Oct4 and Sox2 to distinct Sox/Oct motifs illustrated the ability of FP-EMSA to readily reveal co-operative DNA binding. Incubation of Oct4 and Sox2 with the *Nanog* Sox/Oct motif resulted in almost all of the Oct4 and Sox2 proteins being present in an Oct4–Sox2–DNA complex band with little Sox2–DNA or Oct4-DNA detected (Fig1C). In contrast, binding of Oct4 and Sox2 to DNA containing a 3-bp insertion between the Sox and Oct binding sites resulted in persistent, intense monomer band formation by both Sox2 and Oct4 (Fig1D).

### Comparative quantitative binding of Oct4 and Sox2 to the Sox/Oct and PORE motifs

**Figure 2:**
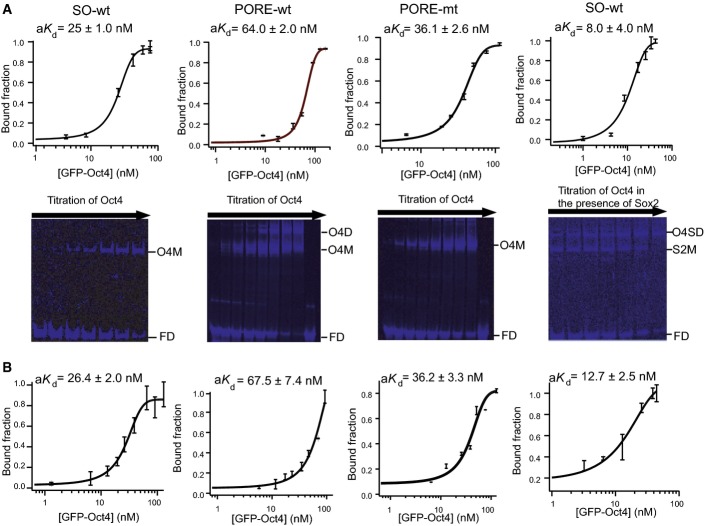
Affinity of Oct4 binding to Sox/Oct and PORE motifs Titrations of GFP-Oct4 are shown from left to right with the *Nanog* Sox/Oct motif, the PORE-wt, the PORE-mt (a DNA probe in which one of the 2 binding sites in the PORE palindrome is mutated) and the *Nanog* Sox/Oct motif in the presence of mCherry-Sox2. Plots for quantitation of a*K*_d_ are shown below (*n *=* *3, mean ± SD). O4SD, GFP-Oct4 and Sox2 dimer complex; S2M, mCherry-Sox2 monomer complex; O4M, GFP-Oct4 monomer complex; FD, free DNA.

To extend these qualitative results, we performed quantitative measurements of the apparent dissociation constant (a*K*_d_) for the homodimers formed by Oct4 and the heterodimers of Oct4 and Sox2 on specific *cis* regulatory motifs. To do so, we developed a quantitative titration assay based on FP-EMSA (FigEV2). Binding of Oct4 alone was stronger on the *Nanog* Sox/Oct motif (a*K*_d_ of 25.0 ± 1.0 nM; Fig2A) than on the PORE (a*K*_d_ of 64.0 ± 2.0 nM; Fig2A). Moreover, the affinity of binding of Oct4 to the *Nanog* Sox/Oct motif was increased 2- to 3-fold in the presence of Sox2 (a*K*_d_ of 8.0 ± 4.0 nM; Fig2A) indicative of co-operative binding of Oct4 and Sox2 to DNA. In contrast, comparison of a*K*_d_ of Oct4 for PORE and a variant PORE in which one of the palindromic repeats was mutated indicates that two Oct4 proteins do not co-operatively form a homodimer on the PORE (Fig2A). Overall, Oct4 shows preferential binding to the Sox/Oct motif compared to the PORE element, irrespective of whether a monomer or dimer is binding to the PORE element. Importantly, the a*K*_d_s determined by FP-EMSA were in good agreement with the aK_d_s determined by the independent method of FCS (Fig2B) 35, 36, 37, confirming the utility of the FP-EMSA approach.

**Figure:**
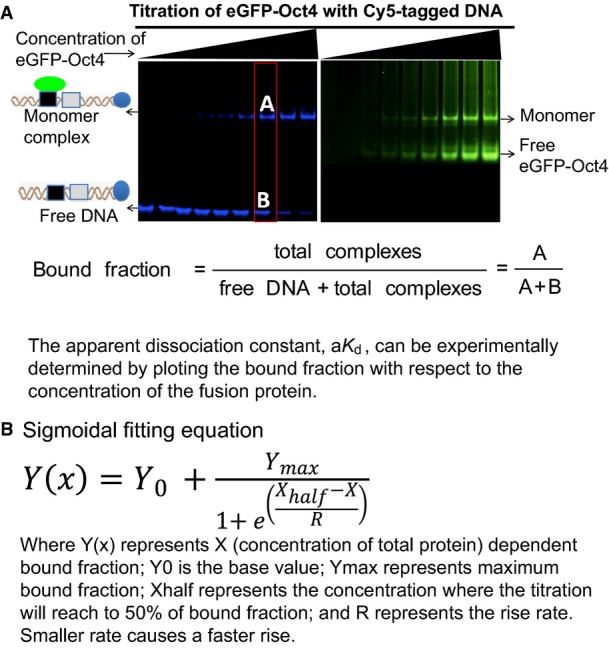
Quantification of a*K*_d_ by FP-EMSA Blue panel: DNA; green panel: protein detection. For quantification purpose, only the blue panel needs to be taken into consideration. The bound fraction was calculated according to the equation provided.

Sigmoid fitting function used to fit the bound fraction versus total protein concentration plot. Source data are available online for this figure.

Quantitation by FCS was performed independently (*n *=* *3, mean ± SEM). All oligonucleotide sequences are in Appendix Table S1. Source data are available online for this figure.

### Sox2 binds synergistically to the Sox/Oct motif with Oct4 but not Oct6

**Figure 3:**
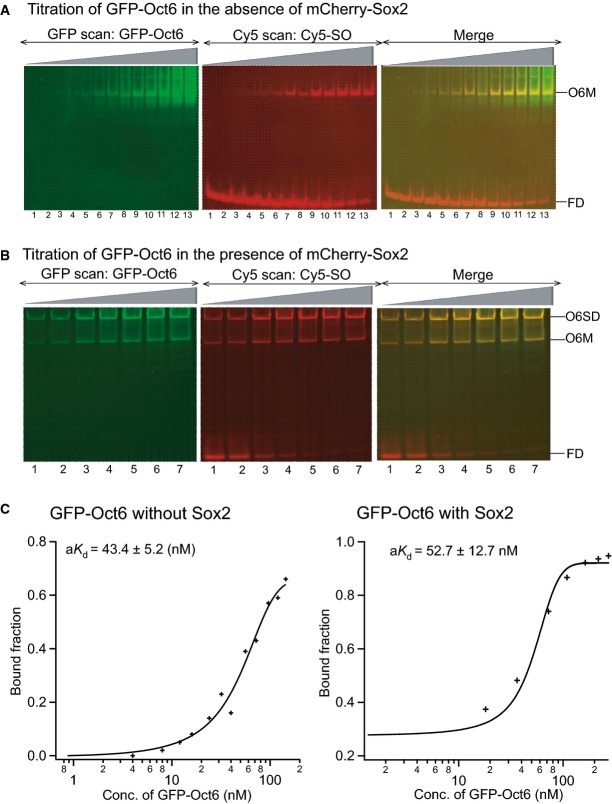
Sox2 has no influence on the binding of Oct6 to the Sox/Oct motif A, B FP-EMSA analysis of titrations of GFP-Oct6, binding to the *Nanog* Sox/Oct oligonucleotide (10 nM) in the absence (A) or the presence (B) of 132 nM mCherry-Sox2 (*n *=* *2). O6SD: GFP-Oct6-mCherry-Sox2 dimer, O6M: GFP-Oct6 monomer complex, FD: free DNA (*n *=* *2).

The ability of Sox2 to stimulate ternary DNA complex formation by Oct4 or Oct6 on the *Nanog* Sox/Oct motif was next compared. Oct4 and Oct6 have similar abilities to form monomers on the *Nanog* Sox/Oct motif in the absence of Sox2 (Figs3A and EV3D). However, in the presence of Sox2, Oct4 and Oct6 behaved drastically different. Oct6 and Sox2 bind to the *Nanog* Sox/Oct motif as a combination of individual monomers and heterodimers, with monomers present even at an Oct6 concentration of 270 nM (Fig3). Moreover, the a*K*_d_ of Oct6 for DNA was unaffected by the presence of Sox2 (Fig3C). In contrast, at 50 nM Oct4, Sox2 is driven almost exclusively into heterodimer formation on the *Nanog* Sox/Oct motif (Fig EV3). Together, these results suggest that at the same concentrations, Sox2 has little influence on Oct6, while it strongly stimulates DNA binding by Oct4.

**Figure:**
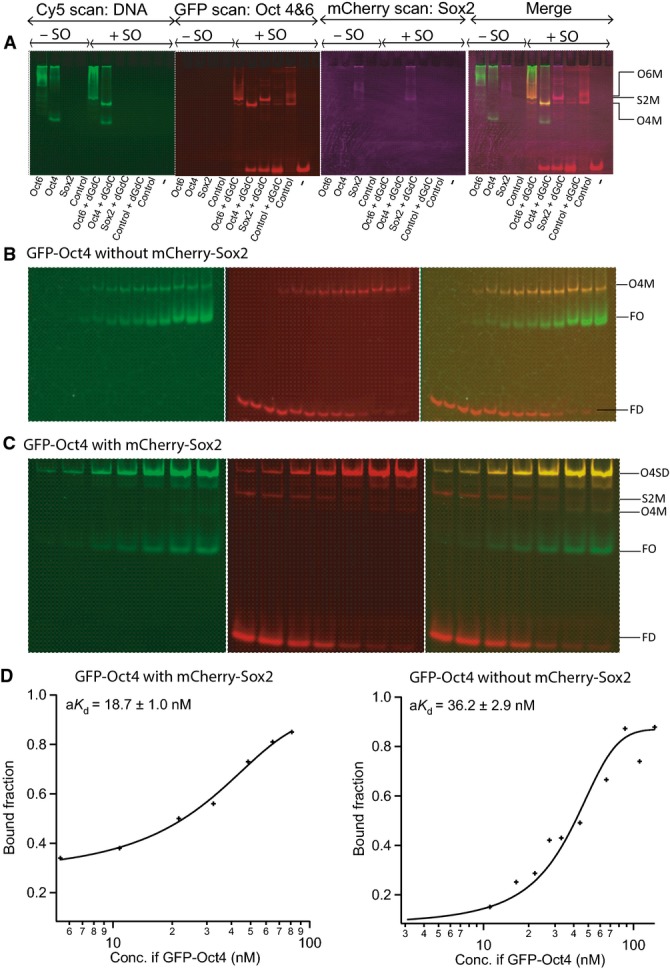
Control experiments for the titration of Oct6 in the presence and absence of Sox2 A Confirmative monomeric band was observed by studying proteins in the absence (−SO) or the presence (+SO) of the *Nanog* Sox/Oct sequence. Control is lysate from untransfected cells. dGdC is included as a non-specific competitor.

B, C FP-EMSA analysis of titrations of GFP-Oct4, binding to the *Nanog* Sox/Oct oligonucleotide (10 nM) in the absence (B) or the presence (C) of 132 nM mCherry-Sox2.

D Bound fractions observed from titrations shown in (B, C) were plotted against the respective concentration of Oct4 for quantifying the a*K*_d_ of those titrations. Data information: O4SD: GFP-Oct4-mCherry-Sox2 dimer complex, O6M: GFP-Oct6 monomer complex, S2M: mCherry-Sox2 monomer complex, O4M: GFP-Oct4 monomer complex, FO: free GFP-Oct4, FD: free DNA. *n *=* *2. Source data are available online for this figure.

C Plots for quantitation of a*K*_d_ of GFP-Oct6 in the presence or absence of Sox2 are shown. Source data are available online for this figure.

### Oct6 homodimerises more efficiently than Oct4 on palindromic motifs

**Figure 4:**
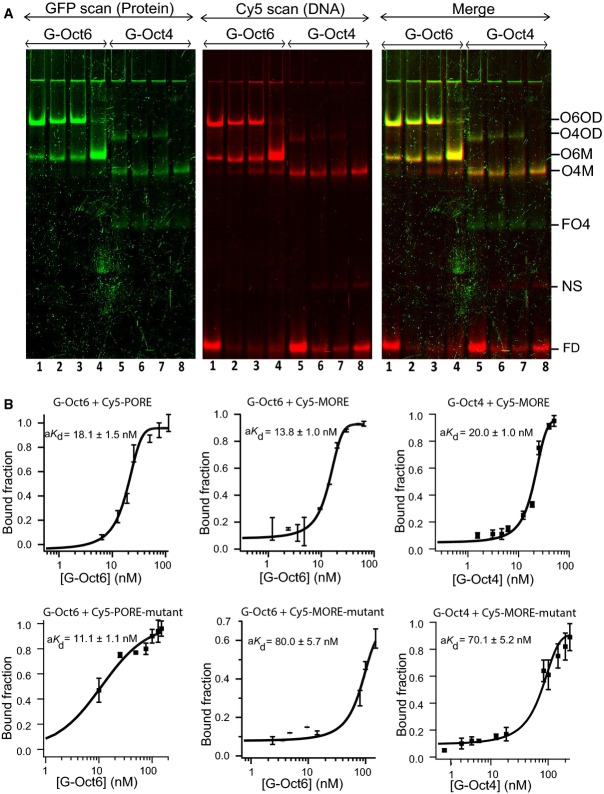
Comparative affinities of Oct4 and Oct6 for palindromic motifs Homodimerisation of GFP-Oct6 (lane 1–4) or GFP-Oct4 (lane 5–8) assessed by FP-EMSA. Green: DNA, red: protein. Lanes 1: MORE-wt; lanes 2: PORE-wt with Cy5 tagged to the 3′ end; lanes 3: PORE-wt with Cy5 tagged to the 5′ end; lanes 4: PORE-mt. O6OD: GFP-Oct6-GFP-Oct6 dimer, O4OD: GFP-Oct4-GFP-Oct4 dimer, O6M: GFP-Oct6 monomer, O4M: GFP-Oct4 monomer, FO6: free GFP-Oct6, FO4: free GFP-Oct4, NS: non-specific binding, FD: free Cy5-tagged DNA. *n *=* *2.

The ability of Oct4 and Oct6 to form homodimers on different palindromic motifs was next compared. Qualitative (Fig4A) and quantitative (Figs2 and 4B) analyses showed that Oct6 has a stronger affinity than Oct4 for palindromic motifs. On the PORE, Oct6 has a ∼3-fold stronger affinity than Oct4 (Oct6, a*K*_d_ = 18.1 ± 1.5 nM; Oct4, a*K*_d_ = 64.1 ± 2.0) (Figs2 and 4B), whereas on MORE, the increased affinity of Oct6 compared to Oct4 was more modest (Oct6, a*K*_d_ = 13.8 ± 1.0 nM; Oct4, a*K*_d_ = 20.0 ± 1.0 nM) (Fig4B). Notably, both Oct6 and Oct4 form homodimer complexes more efficiently on the MORE than the PORE motif (Fig4).

Plots for quantitation of a*K*_d_ of GFP-Oct6 or GFP-Oct4 for wild-type (top) or mutant palindromes (bottom) are shown. *n *=* *3, mean ± SEM. Source data are available online for this figure.

### Distinct sequences in chromatin are bound by ESC- and NSC-specific POU-TFs

**Figure 5:**
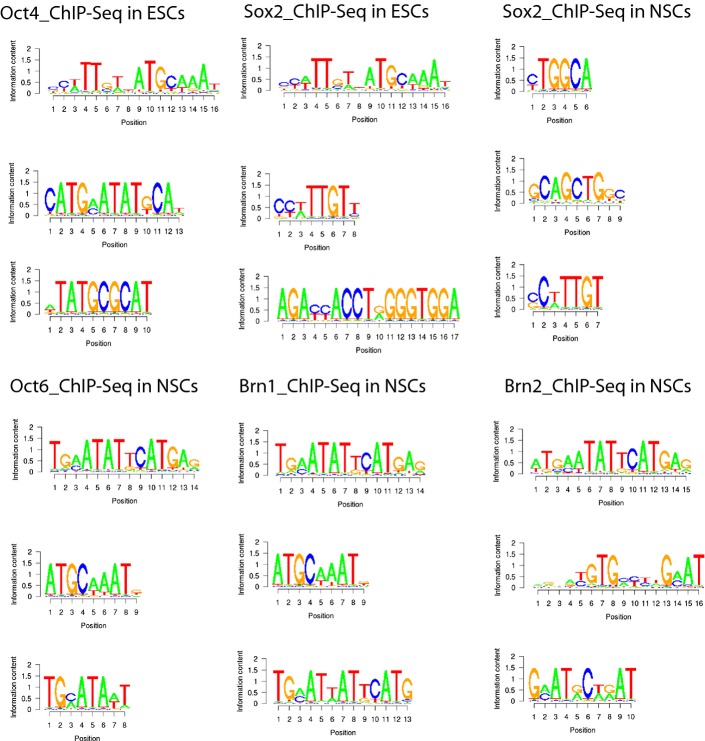
The most enriched TF binding sequences in ESC and NSC chromatin The top *de novo* sequence motifs (based on enrichment) detected by *XXmotif* (see Materials and Methods) in ChIP-Seq data for Oct4 in ESCs, Sox2 in ESCs, Sox2 in NSCs (upper panel), Oct6, Brn1 and Brn2 in NSCs (lower panel). The most enriched motif in Oct4 and Sox2 ChIP-Seq data from ESCs is a composite Oct/Sox motif. This motif is not recovered from ChIP-Seq of NSCs with Sox2 or the multiple POU-TFs shown (see also a more extensive list in Appendix Fig S3). Rather, the top motif recovered from Oct6, Brn1 and Brn2 ChIP-Seq of NSCs is the MORE. Sox2 ChIP recovered a Sox motif as 2^nd^ and 3^rd^ most enriched sequence from ESCs and NSCs, respectively. Shown are the position frequency matrices visualised by WebLogo.

The foregoing analyses indicate different modes of DNA binding by Oct4 and Oct6 in the presence of Sox2. To assess whether this resulted in distinct sequence binding by POU-TFs in Sox2-expressing cells, global localisation of Sox2, Oct6 and the related POU-TFs, Brn1 and Brn2 on chromatin was analysed by ChIP-Seq of the NSC line NS5 38. The XXmotif algorithm was then used to determine putative recognition sequences (“motifs”) within MACS-called peaks of Oct6, Brn1, Brn2 and Sox2 in the ChIP-Seq data. These motifs were compared to putative binding motifs for Oct4 and Sox2 in ESCs using publicly available ChIP-Seq data. As expected, the most enriched motif for both Oct4 and Sox2 in ESCs was the Sox/Oct motif (Fig5 and Appendix Fig S3), in agreement with earlier results obtained using distinct *de novo* motif search tools 24, 39. However, in NSCs, the most enriched motif in the global ChIP-Seq data sets for each of Oct6, Brn1 and Brn2 was not a Sox/Oct motif, but rather a MORE motif (Fig5 and Appendix Fig S3). Neither was the Sox/Oct motif enriched in the Sox2 ChIP-Seq data from NSCs, although a consensus Sox motif was among the top three motifs found (Fig5 and Appendix Fig S3). Other top motifs for both Sox2 and NSC-specific POU-TFs showed no resemblance to one another (Fig5 and Appendix Fig S3). Recent analysis of ChIP data from ESCs undergoing neural differentiation reported extensive co-localisation of Sox2 and Brn2 in neural progenitor cells (NPCs) 30. That analysis showed an overlap in the sequences recovered by ChIP for Sox2 and Brn2. However, comparison of the distances between ChIP peaks for Sox2 and Brn2 in NPCs or between Sox2 and Oct4 in ESCs indicated that Brn2 and Sox2 were more loosely associated with one another throughout the NPC genome than Oct4 and Sox2 are in the ESC genome 30. We re-analysed the ChIP-seq peak data of Lodato *et al* to determine which sequences were most frequently bound by Brn2 and Sox2. In NPCs, the Sox/Oct motif was not evident. Rather, the top motif recovered by Brn2 was a MORE, with a Sox motif among the top motifs recovered by Sox2 (FigEV4). These results provide independent validation of our finding that class III POU-TFs bind to the NSC chromatin predominantly via the MORE palindrome.

**Figure:**
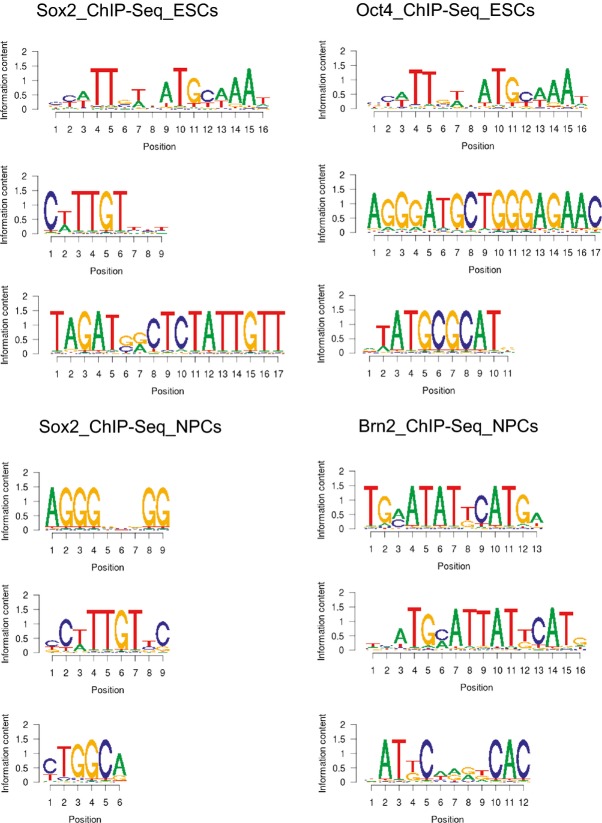
*De novo* motif discovery from the published ChIP-Seq data (Lodato *et al*, 2013) The top *de novo* sequence motifs (based on enrichment) detected by *XXmotif* (see Materials and Methods) in ChIP-Seq data for Oct4 in ESCs, Sox2 in ESCs (upper panel), Sox2 in NPCs and Brn2 in NPCs (lower panel). The top 3 enriched motifs for each are shown by the position frequency matrices visualised by WebLogo.

### A competitive binding assay confirms preferential heterodimerisation of Oct4 and homodimerisation of Oct6

**Figure 6:**
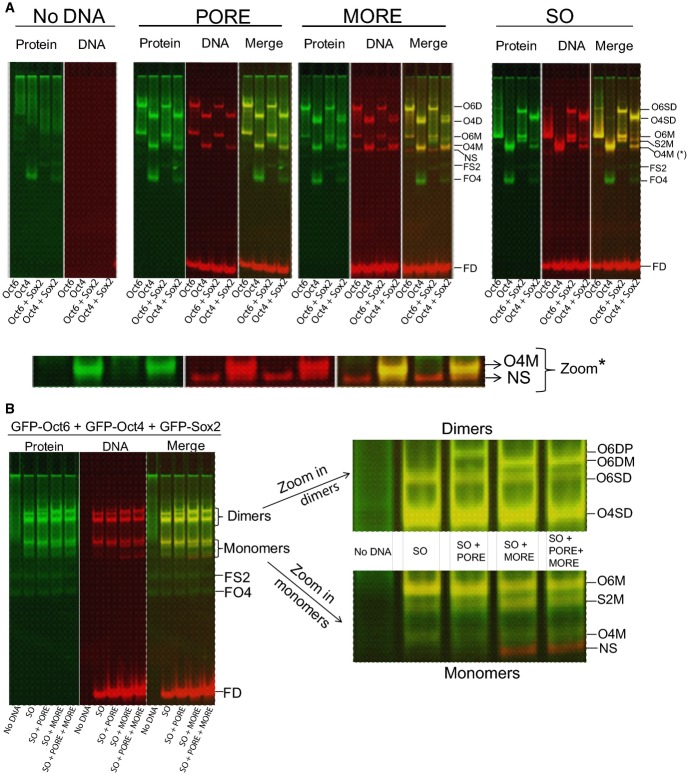
Competitive assay of Oct4 and Oct6 binding to DNA motifs GFP-tagged Oct4, Oct6 and Sox2 bind with different motifs. Panels 1–4 show no DNA, PORE, MORE or the *Nanog* Sox/Oct motif. O6SD: GFP-Oct6-GFP-Sox2 dimer, O4SD: GFP-Oct4-GFP-Sox2 dimer, O6D: GFP-Oct6 homodimer, O4D: GFP-Oct4 homodimer, O6M: GFP-Oct6 monomer, O4M: GFP-Oct4 monomer, NS: non-specific band, FS2: free GFP-Sox2, FO4: free GFP-Oct4, and FD: free Cy5-tagged DNA. *n *=* *2.

The results of the foregoing analyses suggested that Oct6 preferentially bound to the MORE even in the presence of Sox2, while Oct4 co-operatively binds with Sox2 on the Sox/Oct motif. This hypothesis was tested directly using a competitive binding assay. First, control experiments with individual proteins in the presence of single motifs established the position of individual band shifts for Oct4 and Oct6 either alone or in combination with Sox2 (Fig6A). Next, Oct4, Oct6 and Sox2 were incubated in combination with an equimolar concentration of MORE, PORE and Sox/Oct DNAs. In this case, the most prominent band was that formed by Oct4 and Sox2 on the Sox/Oct motif. The Sox/Oct motif also formed a complex with Sox2 and Oct6. However, this was proportionally less than the Oct6 dimer formed on the MORE. Under these competitive conditions, Oct6 binding to the PORE could not be detected (Fig6B). This experiment establishes that the preferential binding order for Oct6 is to form homodimers on the MORE, heterodimers on the *Nanog* Sox/Oct motif and then homodimers on the PORE, while Oct4 prefers to form a heterodimer with Sox2 on the *Nanog* Sox/Oct motif. These results are in accord with the relative a*K*_d_s and provide a biochemical explanation for the preferential recovery of the MORE motif from class III POU-TF ChIP-Seq analyses of NSCs (Fig5).

To test the priority in dimer formation, cell lysates containing Oct6, Oct4 and Sox2 were mixed with all three DNA motifs and complexes assessed by FP-EMSA. The oligonucleotides incubated in each FP-EMSA are indicated at the bottom; dimer and monomer zones have been enlarged on the right. O6DP: GFP-Oct6 homodimer on PORE, O6DM: GFP-Oct6 homodimer on MORE, S2M: GFP-Sox2 monomer. *n *=* *2. Source data are available online for this figure.

### Class III POU-TF-associated MORE elements in NSCs

While target genes of Oct4 have been widely described 40, 41, few known targets of Oct6 exist in NSCs 25, 42, 43. Given the preference for Oct6 homodimerisation on the MORE, the identification of discrete instances of natively occurring MOREs within the ChIP-Seq data set was sought. The top motif identified in the *de novo* motif discovery is a variant (ATGAATATTCAT) of the conventional MORE (ATGCATATGCAT) (Fig5). Brn2 and Pou class IV factors have previously been identified to homodimerise on this variant MORE *in vitro* 44. In addition, within the discovered *de novo* motifs, there was evidence of MORE+1, a MORE with a single-base insertion between the two half-sites that Oct4 and Brn2 have been shown to bind to *in vitro* 26, 44. The MORE+1 motif (3^rd^ motif in Brn1 of NSCs, Fig5; 2^nd^ motif in Brn2 of NPCs, Fig EV4) suggests the class III POU-TFs may bind this MORE variant *in vivo*.

**Figure 7:**
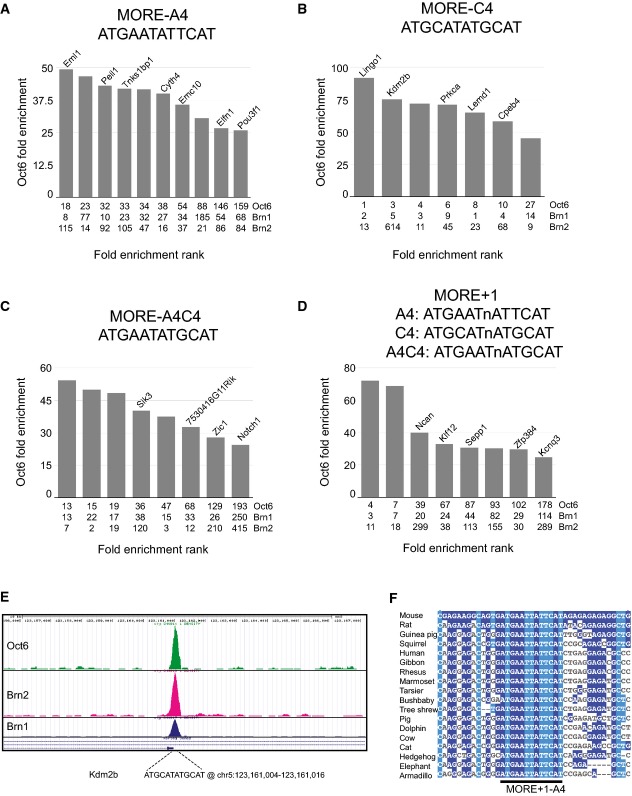
Top Oct6-bound MOREs in NSCs A–D The top 200 MACS-defined Oct6 ChIP-Seq peaks were mined for the presence of MORE sequence subtypes A4 (A), C4 (B), A4C4 (C) and MORE+1 (D). Ordering of data across the *x*-axis is based on fold enrichment of Oct6. The rank value position of the peak is shown on the *x*-axis including Brn1 and Brn2. The gene name is given above the bar for the nearest transcription start site (TSS) to the MORE, where the TSS is within 100 kb.

MORE-like elements identified within the top 5% (based on fold enrichment) Oct6 ChIP-Seq peaks represented a total of two hundred Oct6-bound locations within the NSC genome. Perfect matches to the conventional MORE were found in seven of these, including the very top bound peak (Fig7A). One of these contained 16 bases (CCTCATGCATATGCAT) identical to the MORE oligonucleotide used in our EMSAs. Ten peaks contained perfect matches to the variant MORE found as the top *de novo* motif (Fig7B). This element can be defined as a MORE subtype since, like the conventional MORE, it is a perfect palindrome that, from the ChIP-Seq data, binds Oct6, Brn1 and Brn2. We suggest subtyping nomenclature be defined by the 4^th^ position within the MORE. Thus, the conventional MORE would be MORE-C4 and the subtype first defined *in vitro* by Rhee *et al* 44 would be MORE-A4. Composite elements containing one half-site each of these two MOREs were also identified. Eight instances of such MORE-A4C4 elements were found (Fig7C). Eight elements containing a single-base insertion between half-sites were also identified. These MORE+1 sites were either C4 or A4 subtype or a composite of these (MORE+1-A4C4; Fig7D). No instances of perfect palindromic MORE-G4 and MORE-T4 elements, the later known to bind Pit-1/Pou1f1, were found 45, nor any composite elements using a half-site from these MORE subtypes. In addition, no identifiable Sox/Oct or PORE elements were found in the top 200 Oct6 ChIP-Seq peaks. As 33 of the top 200 ChIP-Seq peaks contain exact identity to MORE elements, with potentially more peaks harbouring subtle variations on these, we conclude that class III POU-TFs predominantly mediate their binding to DNA in NSCs through MORE *cis* architecture.

E ChIP-Seq profiles of Oct6, Brn1 and Brn2 on the *Kdm2b* gene.

F Sequence alignment around the MORE associated with *Kcnq3*.

### Identification of MORE regulated genes

Genes associated by proximity to these Oct6-bound MOREs include epigenetic regulators and factors known to be involved in NSCs and neural development. While its role in NSCs remains unknown, *Lemd1* expression has been reported to be induced by ectopic Brn2 expression 30; this gene contains a MORE-C4 within intron 1 (Fig7B). *Kdm2b*, a polycomb complex regulator 46, contains a MORE-C4 43-bp upstream of its transcription start site (Fig7E). A conserved MORE-A4 is associated with *Pou3f1*, the Oct6-encoding gene (Fig7A). Genes encoding the NSC regulator Notch1 and the neural transcription factor Zic1 have MORE-A4C4 motifs (Fig7C) within 18 and 10 kb of their respective transcription start sites. In a functional test of both our GFP-Oct6 fusion construct and the *Kdm2b* and *Zic1-*associated MORE elements, we found GFP-Oct6 overexpression in ESCs induced the expression of these respective genes (Appendix Fig S1E), in accordance with a previous report of Oct6 induction of *Zic1* 47. *Kcnq3*, which encodes a neuronal ion channel, is associated with a MORE+1-A4 motif that is completely conserved across all eutherian with conservation in the immediately surrounding sequence dropping markedly (Fig7D). This association with neural-related genes in addition to the sequence conservation found within several of these MOREs and exemplified here by *Kcnq3*, presumably through purifying selection, argues strongly for a functional role of MORE-bound class III POU-TFs in NSCs.

## Discussion

### Sox2 and Oct4 favour heterodimer formation on the Sox/Oct motif compared to Sox2 and Oct6

A prevalent model to explain how cell identity is gradually altered during developmental progression proposes that Sox TFs control cellular transitions by switching partners 48, 49. In this study, quantitative EMSAs have been used to investigate DNA binding by ESC- and NSC-specific POU-TFs in the presence and absence of Sox2 and ChIP-Seq has been used to assess motif binding by Sox2 and POU-TFs in ESCs and NSCs. ChIP-Seq analysis shows that in ESCs, the most predominant DNA motif identified in both Sox2 and Oct4 chromatin immunoprecipitates is a composite Sox/Oct motif. Surprisingly, the Sox/Oct motif was not identified by ChIP-Seq of Sox2 or class III POU-TFs in NSCs. Instead, the top motifs recovered from ChIP-Seq analysis of NSCs for Sox2 included a simple Sox motif, with the MORE palindromes being the top motif in analyses of each of the POU-TFs Oct6, Brn1 and Brn2. This suggests that in NSCs, Sox2 and class III POU-TFs do not act co-operatively. As demonstrated by our DNA-binding analyses, the mechanism underlying this preferential recovery is differential affinity for DNA binding sites observed for distinct TFs alone and in combination.

We observed that heterodimer formation on the *Nanog* Sox/Oct motif was favoured by Oct4 in the presence of Sox2 in agreement with previous studies 22, 33. This is due to synergistic binding to DNA by Oct4 and Sox2, which has also been reported at the Sox/Oct motifs of the *Fgf4*, *Utf1* and *Pou5f1* genes. In addition, Oct4 and Sox2 form heterodimers on the Sox/Oct motifs of *Sox2* and *Fbx15* genes although in these cases synergy was not definitively demonstrated 19, 20. Here, using FP-EMSA and FCS, we have been able, for the first time, to measure a*K*_d_s between the relevant individual TFs and DNA and have thereby assessed quantitatively the synergy between Oct4 and Sox2.

In contrast to the situation with Oct4 and Sox2, Oct6 and Sox2 both prefer to bind to the *Nanog* Sox/Oct motif alone rather than as a heterodimer (Fig3). Although Oct6 and Sox2 can bind to the Sox/Oct motif as a heterodimer, this binding is not co-operative. Interestingly, Sox2 and the Oct6-related POU-TF Brn2 can form heterodimer complexes on a non-canonical Sox/Oct motif associated with *Nestin* 50. However, the predominant mode of DNA binding in that study was as Sox2 or Brn2 monomers, suggesting that Brn2, like Oct6, does not have the ability to co-operatively bind DNA with Sox2. This conclusion is also supported by the discovery of an octamer and the absence of a Sox/Oct motif, in the Brn2 ChIP-Seq analysis of neural progenitor cells 30 (Fig EV4) and in the Brn2, Oct6 and Brn1 ChIP-Seq analysis in NSCs (Fig5). These studies underscore the point that co-localisation of TFs in ChIP-Seq data sets does not provide information of the binding mechanisms as it cannot discriminate between synergistic and non-synergistic interactions.

### Dimer formation on MORE is generally stronger than on PORE motifs

Our DNA binding studies suggest that homodimer formation, by both Oct6 and Oct4, is favoured on MORE motifs, relative to PORE motifs. Crystallographic analysis of homodimer formation on the PORE motif demonstrated that the POU_S_ and POU_HD_ domains of each monomer bind across the two DNA strands forming two protein–protein interfaces, with steric repulsion disfavouring efficient homodimer formation 9, 10. This is consistent with the observed a*K*_d_s of Oct4 and Oct6 for the PORE motif and for a variant PORE motif with mutations in one of the two palindromes. However, a similar situation would not be expected for MORE motifs, as the subunit packing for complex formation differs from that seen on PORE motifs 26, 28. Crystallography of the MORE complex illustrates that the POU_S_ and POU_HD_ domains of each monomer bind on one face of the DNA helix and are stabilised by side chain interactions favouring strong homodimer formation 26, 28. Our DNA affinity measurements provide biochemical support for the different mechanisms responsible for the formation of complexes on MORE and PORE elements.

### Sox2 determines the fate of complex formation for Oct4, but has little influence on Oct6

Oct4 binds similarly well to the Sox/Oct motif and to the MORE motif in the absence of Sox2. However, in the presence of Sox2, Oct4 preferentially binds to the Sox/Oct motif and together Oct4 and Sox2 form stable heterodimers that drive the expression of pluripotency genes 22, 33, 51. Without Sox2, Oct4 forms a stable homodimer, which can facilitate the expression of a different set of genes. For example, cells in the inner cell mass of the blastocyst that transiently express high Oct4 levels can regulate *Spp1* by the formation of PORE homodimers 9. On the other hand, Oct6 shows a higher tendency to form homodimers whether or not Sox2 is present. Although Oct6 can form a Sox2 heterodimer on the *Nanog* Sox/Oct motif with Sox2, this does not occur co-operatively. Therefore, although Sox2 is present in both ESCs and NSCs, Sox2 does not influence Oct6 DNA binding in NSCs. In contrast, Oct4 preferentially forms heterodimers with Sox2 in ESCs. These observations were upheld on a genome-wide level by our ChIP-Seq studies. Taken together, these results suggest that the mode of DNA binding by the NSC-specific POU-TFs Brn1, Brn2 and Oct6 is not directly influenced by Sox2 and therefore differs markedly from that of Oct4 in the context of chromatin in living cells. While we cannot exclude the possibility of a different TF partnering with Sox2 in NSCs, our results would indicate that class III POU-TFs do not and thus should not be considered as players in Sox–partner codes in cell specification 48.

Finally, as our study reveals novel modes of protein–DNA interactions, it not only explains the difference in the motif enrichment between ESCs and NSCs, but also offers a molecular mechanism for iPS generation from NSCs. Kim *et al* have shown that Oct4 alone can reprogramme NSCs to iPS 31. Our findings suggest that exogenously expressed Oct4 may interact synergistically with endogenously expressed Sox2 to redirect Sox2 to the Sox/Oct motifs in the proximity of pluripotency genes such as *Nanog*, *Oct4*, *Sox2*, *Utf1* and *Fgf4* 16, 18, 21, 22, which are essential for inducing the pluripotent state in NSCs 3. Although Oct6 and Sox2 are endogenously co-expressed in NSCs, the fact that the synergistic interaction of Sox2 with POU-TFs is Oct4-specific may explain how Oct4 alone can drive iPS generation from NSCs by co-operative interaction with Sox2.

## Materials and Methods

### Cell culture and transfection

Fluorescent protein fusions for analysis by FP-EMSA and FCS were prepared by transfection of CHO-K1 cells (ATCC # CRL-61). Cells were cultured in Dulbecco’s modified Eagle’s medium (DMEM, GIBCO-19600), 10% foetal bovine serum (GIBCO), 1× non-essential amino acids, 2 mM l-glutamine, 1× penicillin/streptomycin at 37°C, 5% CO_2_ and 95% humidity. Transfection of plasmids was carried out by using Lipofectamine 2000 (Invitrogen) according to the manufacturer’s instructions. Twenty-four μg of plasmid was transfected into CHO cells on 55-cm^2^ plates. The cells were collected after 24 h to prepare nuclear cell lysates.

### Fusion protein construction

The fluorescent proteins GFP and mCherry were used to make amino-terminal fusions of mouse Oct4, Oct6 and Sox2 TFs by standard PCR techniques in which GFP-Oct4, GFP-Oct6 and mCherry-Sox2 were connected by the linker Gly-Gly-Ser-Gly. The fused ORF was then expressed from the CAG promoter and linked via an IRES to an appropriate drug selection cassette.

### Functional assessment of Sox2 activity

Sox2CKO cells (1 × 10^7^) 2 were transfected with pPyCAG-Sox2-IH 2, pPyCAG-mCherry-Sox2-IH or pPyCAG-IH 2 using Lipofectamine 3000 according to the manufacturer’s instructions. Twenty-four hours post-transfection, cells were replated at 10^6^/63-cm^2^ dish; 48 h post-transfection, cells were cultured in the presence of hygromycin B (100 μg/ml) and 1 μM 4-OH tamoxifen (Sigma, H7904) for 12 h to delete the endogenous *Sox2* allele. Cells were cultured in 100 μg/ml hygromycin B for a further 9 days before alkaline phosphatase staining (Sigma, 86R). The rescue efficiency was determined by normalisation of colony number per plasmid with respect to that of pPyCAG-Sox2-IH.

### Functional assessment of Oct4 activity

ZHBTc4.1 cells (1 × 10^7^) 3 were transfected with pPyCAG-Oct4-IP 4, pPyCAG-GFP-Oct4-IP or pPyCAG-IP 4 using Lipofectamine 3000 according to the manufacturer’s instructions. Twenty-four hours post-transfection, cells were replated at 10^6^/63-cm^2^ dish in the presence of 1 μg/ml puromycin and doxycycline (dox) (1 μM) (Sigma). Cells were cultured in 1 μg/ml puromycin for 7 days before alkaline phosphatase staining (Sigma, 86R). The rescue efficiency was determined by normalisation of colony number per plasmid with respect to that of pPyCAG-Oct4-IP.

### Functional assessment of Oct6 activity

E14Tg2a cells (5 × 10^5^) were transfected with pCAG-GFP-Oct6-IN using Lipofectamine 3000 according to the manufacturer’s instructions. Twenty-four hours post-transfection, cells were replated at 10^6^/9.5-cm^2^ dish in the presence of 200 μg/ml G418 and media replenished every 2 days. Cells were harvested 6 days post-transfection and RNA extracted using RNase plus mini kit (Cat number- 74134, QIAGEN). RNA was reverse-transcribed with SuperScript III (Invitrogen) and qPCR performed in 384-well plates on a 480 LightCycler (Roche) system using LightCycler 480 SYBR Green I Master (Roche). Primer sequences are listed in Appendix Table S2.

### Nuclear lysate preparation

Nuclear extracts were prepared essentially as described 5. Briefly, cells grown on 55-cm^2^ plates were harvested with 1 ml phosphate-buffered saline followed by trypsin–EDTA treatment. The cell pellet was resuspended in 400 μl pre-chilled hypotonic buffer (10 mM HEPES-KOH pH 7.9, 15 mM MgCl_2_, 10 mM KCl, 0.5 mM DTT, 0.2 mM PMSF) and then incubated at 4°C for 10 min, vortexed for 10 seconds and centrifuged. The cell pellet was resuspended in 0.6 pellet volume pre-chilled hypertonic buffer (20 mM HEPES-KOH pH 7.9, 1.5 mM MgCl_2_, 420 mM NaCl, 0.2 mM EDTA, 0.5 mM DTT and 0.2 mM PMSF), incubated on ice for 20–25 min and centrifuged at 4°C. The supernatant was then preserved at −80°C until further use.

### Concentration measurement of fusion proteins by fluorescence correlation spectroscopy (FCS)

The FCS set-up is based on a commercial Laser Scanning Confocal Microscope (FV300, Olympus, Singapore) with a water immersion objective (60×, NA1.2, Olympus) coupled to a custom-built FCS module 52. In the FCS module, the fluorescence light from the confocal microscope was split by a dichroic mirror (560DRLP, Omega Optical, Brattleboro, VT, USA) into two detection channels. For excitation at 488, 543 and 633 nm, emission light was filtered by 510AF23, 593AF40 and 670AF60 bandpass filters (Omega Optical), respectively. A standard calibration approach was performed to determine the absolute concentration of fluorescent protein-tagged TFs in the nuclear lysate 53, 54. Dyes with known diffusion coefficients were employed in order to determine the confocal volume. All measurements were performed at room temperature. For measuring the concentration of GFP-Oct4 and GFP-Oct6, the calibration was carried out with 5 nM fluorescein (diffusion constant, D = 4.25 × 10^−6^ cm^2^ s^−1^) being excited by a 488 nm laser line at 30 μW. For mCherry-Sox2, the calibration was performed with a 543-nm laser line at 30 μW using 5 nM of Rhodamine 6G (D = 4.14 × 10^−6^ cm^2^ s^−1^). Cy5-labelled DNA calibrations were performed using 5 nM Cy5 (D = 3.6 × 10^−6^ cm^2^ s^−1^). The confocal volumes were 0.58, 0.72 and 0.82 × 10^−15^ L, respectively. Correlations were computed online by a hardware correlator (Flex02-01D, Correlator.com, Bridgewater, NJ, USA) and were fitted by a model for free diffusion in solution and a triplet using a self-written program in Igor Pro 6 (WaveMetrics, Lake Oswego, OR, USA). Absolute concentrations were calculated as compared to the standard dyes and are given in nM.

### Electrophoretic mobility shift assay (EMSA)

EMSA was performed using 37-bp double-stranded Cy5-labelled oligonucleotides (Sigma) containing Sox/Oct or PORE/MORE motif sequence (Appendix Table S1). Each binding reaction consisted of 0.5 μl of 2 μM Cy5-tagged DNA motif, 1.5 μl poly (deoxyguanylic–deoxycytidylic) acid sodium salt (2 μg/μl dGdC) (Sigma), 1 μl of 80% glycerol in buffer C (60% of 20 mM HEPES pH 7.9, 10 mM KCl, 1.66 mM DTT, 1% protease inhibitor mix (Sigma), 0.83 mM EDTA) and 9 μl of fusion protein extract in buffer C for a final volume of 12 μl. In titration experiments using less fusion protein extract, volumes were adjusted to 12 μl with buffer C. For titration experiments, DNA concentration was 5 nM, unless otherwise indicated. Binding reactions were incubated (4°C, 30 min), run on a 6% polyacrylamide gel (300 V, 2 h) in 0.5× Tris–borate–EDTA and visualised on a Typhoon 910 PhosphorImager (Amersham Biosciences) or a FLA5000 image analyser (FUJIFILM). A fluorescent protein-based EMSA (FP-EMSA) approach was also developed to visualise both fluorescence protein and DNA. This improvement is also economical as it allows untagged oligonucleotides to be used to visualise protein–DNA complexes. The lasers and filters were as follows for the different fluorophores: Cy5 (λ_ex_ = 633 nm; λ_em_ = 670 nm), laser: 633 nm, filter: 670 nm (BP); GFP (λ_ex_ = 488 nm; λ_em_ = 510 nm), laser: 488 or 473 nm, filter: 510 (BP); mCherry (λ_ex_ = 587 nm; λ_em_ = 610 nm), laser: 532 nm, filter: 610 (BP). All DNAs used for EMSA are listed in Appendix Table S1.

### Determination of apparent dissociation constant (a*K*_d_) by EMSA and FCS

Nuclear extract containing fusion protein of known concentration was titrated with Cy5-tagged DNA oligos until the titration reached saturation level. After incubation (30 min, 4°C), reactions of the different titration points were run on an EMSA gel. Individual band intensities were quantified using IQ-quant software and bound fractions of different titration points were calculated (FigEV2A). An empirical sigmoid plot was used to fit the bound fraction plot (FigEV2B). The apparent dissociation constant, a*K*_d_, is the concentration of protein required for a 50% bound fraction. Independent FCS-based binding assays were also applied. To determine the a*K*_d_, autocorrelation curves were fitted with a 3D-2particle-1triplet model using Igor Pro 6.0 as described 37 to determine the bound fraction (Appendix Fig S2). The two independent techniques gave similar a*K*_d_ measurements, thus validating the EMSA-based quantification.

### ChIP-Seq and ChIP-Seq data analysis

ESC-derived NS5 NSCs were cultured as described 38. ChIP was performed as described 55. The antibodies (previously tested and validated for ChIP experiments) that were used were as follows: Sox2, Y17 polyclonal antibody (sc-17320; Santa Cruz Biotechnology) 22; Oct6 and Brn1, rabbit polyclonals (kind gifts from Dies Meijer, Edinburgh); Brn2, Santa Cruz Goat C-20 (sc-6029). Reads were mapped to the mm8 version of the mouse genome. Peaks were called from ChIP-Seq data using MACS 56 and including a background control (input DNA). ChIP-Seq data are deposited in GEO under accession GSE69859, and MACS-called peaks are in Supplementary Table EV1.

### Motif discovery

To identify enriched sequence patterns (motifs) in the putative binding sites (peaks) discovered in the ChIP-Seq analysis, we used a local installation of XXmotif (version 1.6, parameters: –zoops –type ALL -g3 –merge-motif-threshold LOW – batch – revcomp) on the complete set of peak sequences of Sox2, Oct6, Brn1 and Brn2 in NSCs (this study), as well as Oct4 and Sox2 in ESCs 24 (peaks obtained from http://lgsun.grc.nia.nih.gov/CisFinder), as well as Oct4/Sox2 in ESCs and Brn2/Sox2 in NPCs 30. To exclude non-specific sequence patterns, we used DNaseI hypersensitive sites from the ENCODE project (http://genome.ucsc.edu/ENCODE) as a negative control set (parameter:–negSet).

### Identification of Oct6-bound MORE elements in NSCs

The top 5%, based on fold enrichment over control, of Oct6 NSC ChIP-Seq peaks identified by MACS were input into the CisFinder tool 39. The CisFinder output file containing the sequences matching any of the top 25 *de novo* motifs identified by CisFinder was then manually analysed for the presence/absence of sequence elements of interest. Sequence conservation within elements and the position with respect to nearest transcription start site were determined using the UCSC Genome Browser.

## Supporting Information

**Appendix**

**Expanded View Figures PDF**

**Table EV1**

**Review Process File**

**Source Data for Figure 6**

**Source Data for Expanded View and Appendix**
