# Supplementary material for: Selective influence of Sox2 on POU transcription factor binding in embryonic and neural stem cells
Source: EMBO Rep. 2015 Sep 2;16(9):1177–91. doi: 10.15252/embr.201540467 (PMC4576985; doi:10.15252/embr.201540467)
Supplement: Supplementary file 1 [file embr0016-1177-sd1.pdf]

Selective influence of Sox2 on POU transcription factor binding in embryonic and neural stem cells

Tapan Kumar Mistri et al. 2015

Table of contents:

**Content**	**Page(s)**
Appendix Figures S1-S3 and legends	2-5
1. Functional characterization of fluorescent protein fusions of Oct4, Sox2 and Oct6	2-3
2. Comparison of theoretical models for best fit to FCS data	4
3. Enriched Sox2 and POU TF motifs in ChIP-Seq data	5
Appendix Table S1: Oligonucleotides for analysis of Sox/Oct, PORE, and MORE motifs	6
Appendix Table S2: Oligonucleotides used for qPCR	7
References	8

Appendix Figure S1

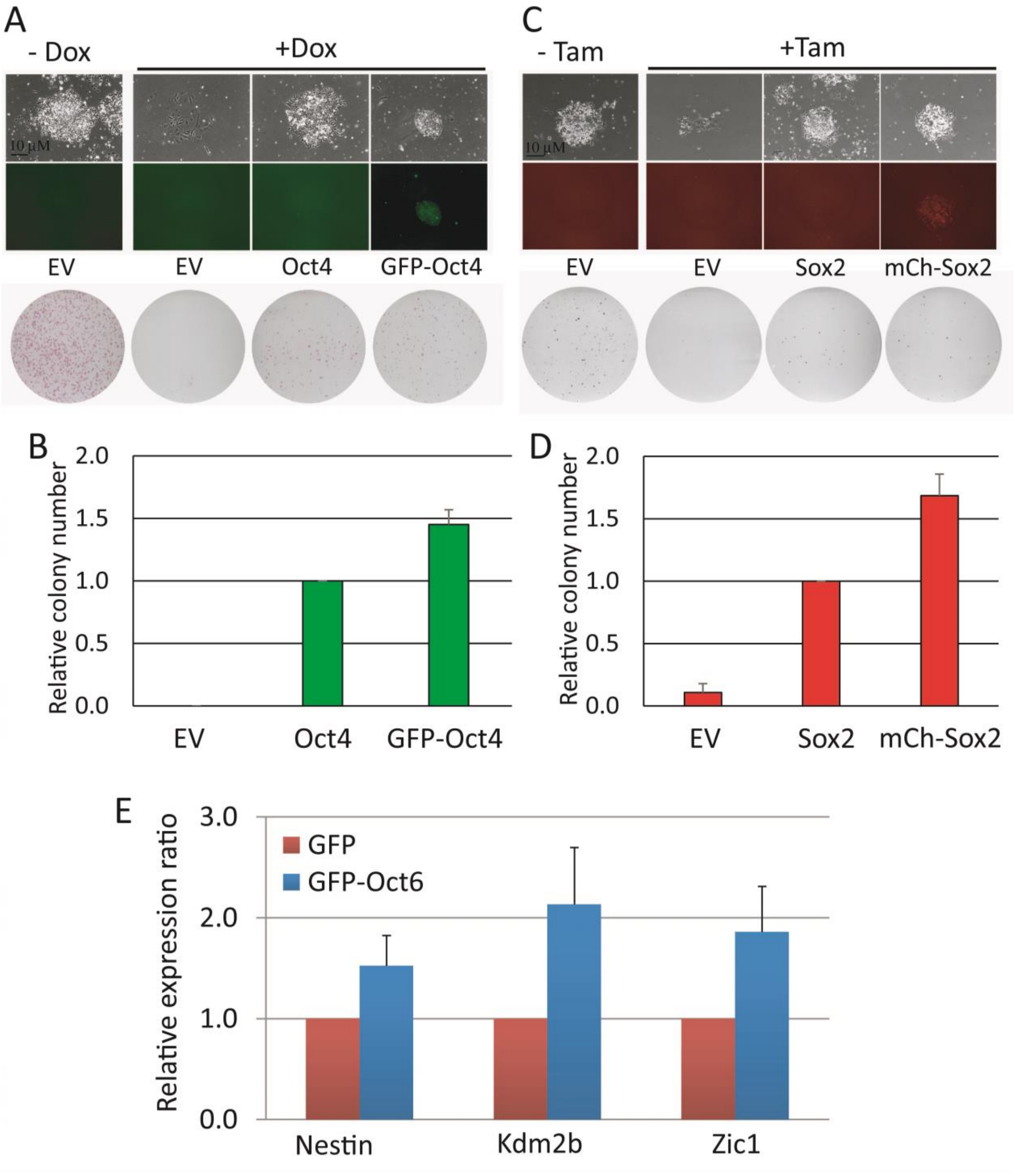

Appendix Figure S1: Functional characterization of fluorescent protein fusions of Oct4, Sox2 and Oct6. (A) ZHBTc4 ES cells, which lack endogenous Oct4 but contain a doxycycline (Dox) suppressible Oct4 transgene, were used to functionally test the GFP-Oct4 fusion protein. In the presents of Dox ES cells differentiate would normally differentiate. Representative morphologies of colonies

formed in the presence of Dox (1μg/ml) and after transfecting with Oct4 (pPyCAG-Oct4-IP), GFP-Oct4 (pPyCAG-GFP-Oct4-IP), or empty vector (pPyCAG-IP) are shown (top). Colonies were stained for alkaline phosphate activity (bottom). (**B**) Quantitation of colony morphologies from alkaline phosphate stained plates shown in (A). (**C**) SCKO cells, in which loxP-flanked *Sox2* can be deleted in response to Tamoxifen (Tam)-inducible Cre-ERT^2 activity and led to differentiation, are assessed for their ES cell colony forming ability after transfection.. Representative morphologies of colonies formed in the presence of Tam (1μg/ml) by cells transfected with Sox2 (pPyCAG-Sox2-IH), mCherry-Sox2 (pPyCAG-mCherry-Sox2-IH), or empty vector (pPyCAG-IH) are shown (top). Colonies were stained for alkaline phosphate activity (bottom). 'mCh' stands for mCherry. (**D**) Quantitation of colony morphologies from alkaline phosphate stained plates shown in (C). (**E**) Relative expression level of two key Oct6 target genes (*Kdm2b* and *Zic1*) and the neural lineage marker *Nestin* by E14Tg2a cells transfected with GFP or GFP-Oct6. Expression level was normalised to *TBP*. All experiments above were performed in triplicate. (n = 3, mean ± SD).

Appendix Figure S2

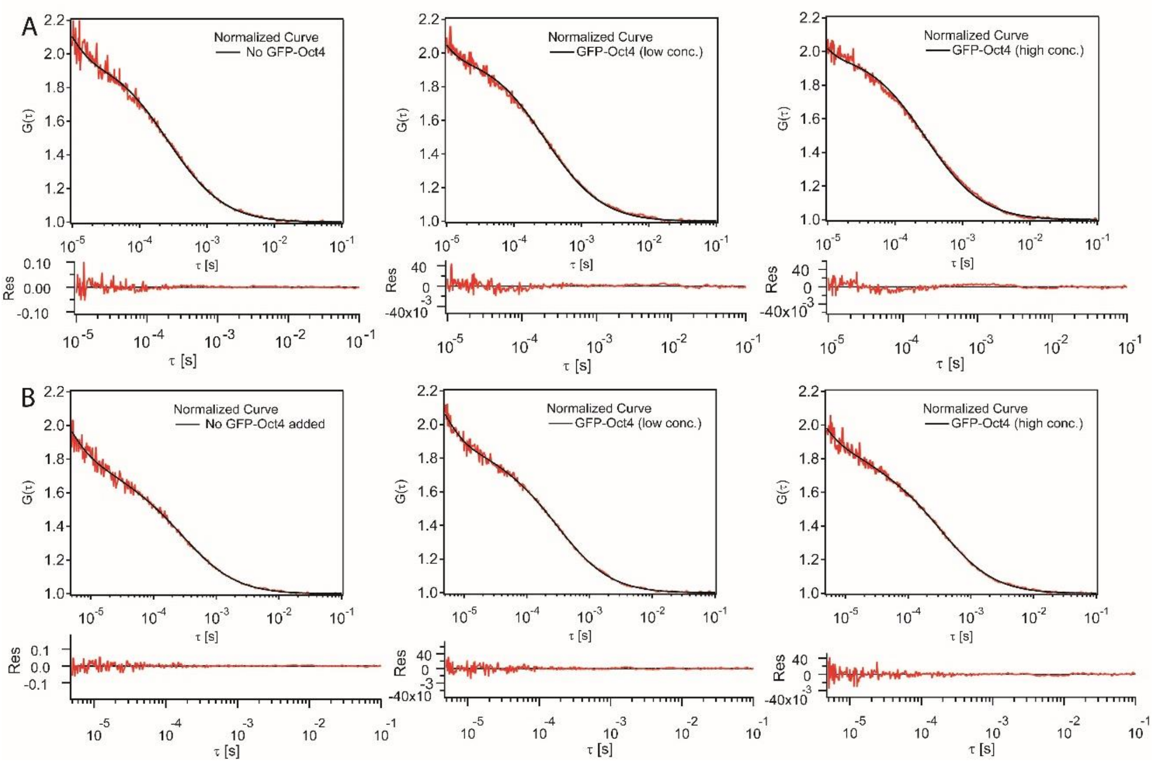

Appendix Figure S2: Comparison of theoretical models for best fit to FCS data. (**A**) A one-component coupled with a triplet state model (3D-1P-1T) used to fit three different ACFs generated from different protein concentration starting from zero (left panel), low (middle) to high (right panel) in a titration reaction with DNA. The attached residual illustrates the goodness of fitting by the model. (**B**) A two-component model (3D-2P-1T) used for fitting the above same ACFs resulting a better residual. The improved residual confirms that 3D-2P-1T is the better model for an ACF generated when protein was added to DNA. Protein-DNA Complex formation is well noticeable from the increased average diffusion time from 273.0 ± 2.9 μs in the absence of protein to 320 ± 5.2 μs at the maximum protein concentration.

Appendix Figure S3

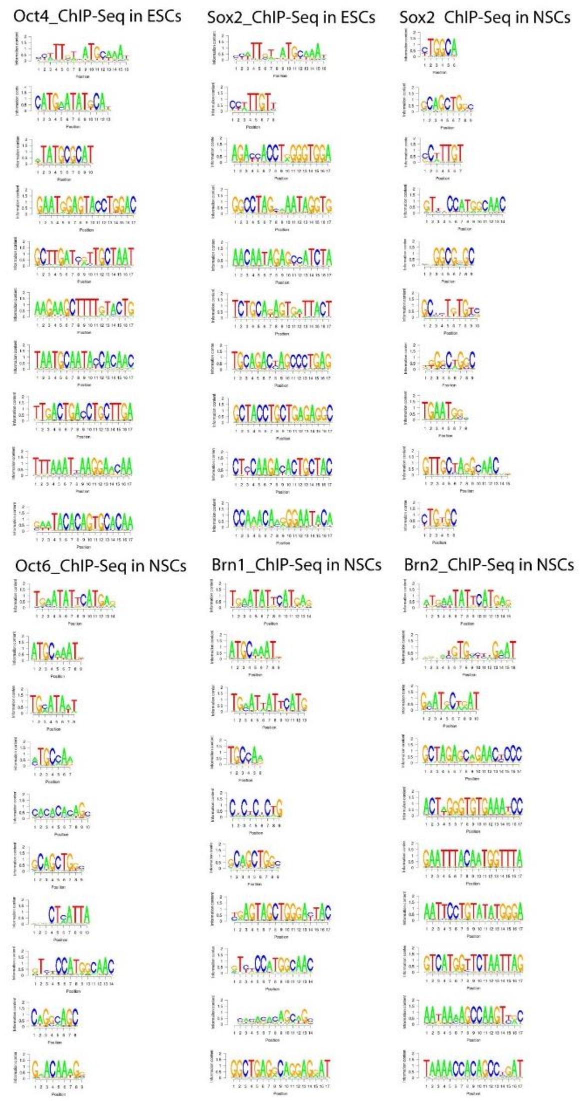

Appendix Figure S3: Enriched Sox2 and POU TF motifs in ChIP-Seq data. The top 10 motifs from Oct4, Sox2, Oct6, Brn1, and Brn2 ChIP-Seq data from E14 ESCs and NS5 NSCs are shown with WebLogo.

Name	Sequence	Figure
SO wt	TCCACCATGGACATTGTAATGCAAAAGAAGCTGTAAG	1, 2, 3, 7, EV1, EV2, EV3
PORE wt	AAGTTAAAATCACATTTGAAATGCAAATGGAAAAGC	1, 2, 4, 7, EV1,
PORE mt	AAGTTAAAATCACtgccaAAATGCAAATGGAAAAGC	1, 2, 4 , 7, EV1,
MORE wt	TGATCACAGCCTCATGCATATGCATATATCGTTGTTG	4, 7
MORE mt	TGATCACAGCCTCATGCATAcctgTATATCGTTGTTG	4
Artificial motif	AAATAATGGAAACTTTGTATTTATGCAAATAAACAAGGTC ATTT	1
fl mut	TcgAccTCACATTTGAAATGCAAAT	
p1 mut	TTAAAATCACAgacGAAATGCAAAT	
p2 mut	TTAAAATCACATTTGAAATGCgtcT	
fl p1 mut	TcgAccTCACAgacGAAATGCAAAT	
fl p2 mut	TcgAccTCACATTTGAAATGCgtcT	EV1C
p1 p2 mut	TTAAAATCACAgacGAAATGCgtcT	
fl p1 p2 mut	TcgAccTCACAgacGAAATGCgtcT	
PORE	TTAAAATCACATTTGAAATGCAAAT	
5L>0	TTAAAATCACATTTGCAAAT	
5L>1	TTAAAATCACATTTGACAAAT	
5L>2	TTAAAATCACATTTGAACAAAT	
5L>3	TTAAAATCACATTTGAAACAAAT	
5L>4	TTAAAATCACATTTGAAATCAAAT	EV1D
PORE	TTAAAATCACATTTGAAATGCAAAT	
L1,3 mut	TTAAAATCACATTTGcAcTGCAAAT	
L3,5 mut	TTAAAATCACATTTGAAgTaCAAAT	
L1-5 mut	TTAAAATCACATTTGcgccaCAAAT	

Appendix Table S1: Oligonucleotides for analysis of Sox/Oct, PORE, and MORE motifs. DNAs used are shown 5'-3'. The red colour indicates the Sox/Oct, PORE, and MORE motifs; mutations are marked by lower case (substitutions) or underling (insertion). The top six DNAs are Cy5 labeled at the 5' end (unless otherwise mentioned in the Figure legend); DNAs used in Figure 2 panels C, D are untagged. fl, P1 and P2 indicate oligonucleotides with mutations in the flanking sequence, palindromic repeat 1 and repeat 2, respectively.

Primers	Sequence
**Nestin-Frw**	CTGCAGGCCACTGAAAAGT
**Nestin-Rev**	TTCCAGGATCTGAGCGATCT
**Zic1-Frw**	AACCTCAAGATCCACAAAAGGA
**Zic1-Rev**	CCTCGAACTCGCACTTGAA
**Kdm2b-Frw**	TCCACCCTGGATGCCTTA
**Kdm2b-Rev**	GCATGGTTACACTTCGGACA
**TBP-Frw**	GGGGAGCTGTGATGTGAAGT
**TBP-Rev**	CCAGGAAATAATTCTGCCTCA

Appendix Table S2: Oligonucleotides used for qPCR. DNAs used are shown 5’-3’. Primers for Nestin and Zic1 were described previously [1]. Primers for Kdm2b were designed using the *ROCHE Universal Probe Library Assay DesignCenter.*

REFERENCES

1. Zhu Q, Song L, Peng G, Sun N, Chen J, Zhang T, Sheng N, Tang W, Qian C, Qiao Y (2014) The transcription factor Pou3f1 promotes neural fate commitment via activation of neural lineage genes and inhibition of external signaling pathways. *ELife* **3:** e02224

2. Gagliardi A, Mullin NP, Ying Tan Z, Colby D, Kousa AI, Halbritter F, Weiss JT, Felker A, Bezstarosti K, Favaro R (2013) A direct physical interaction between Nanog and Sox2 regulates embryonic stem cell selfâ€renewal. *The EMBO journal* **32:** 2231-2247

3. Niwa H, Miyazaki J-i, Smith AG (2000) Quantitative expression of Oct-3/4 defines differentiation, dedifferentiation or self-renewal of ES cells. *Nature genetics* **24:** 372-376

4. Niwa H, Masui S, Chambers I, Smith AG, Miyazaki J-i (2002) Phenotypic complementation establishes requirements for specific POU domain and generic transactivation function of Oct-3/4 in embryonic stem cells. *Molecular and cellular biology* **22:** 1526-1536

5. Andrews NC, Faller DV (1991) A rapid micropreparation technique for extraction of DNA-binding proteins from limiting numbers of mammalian cells. *Nucleic acids research* **19:** 2499
