# Supplementary material for: Selective influence of Sox2 on POU transcription factor binding in embryonic and neural stem cells
Source: EMBO Rep. 2015 Sep 2;16(9):1177–91. doi: 10.15252/embr.201540467 (PMC4576985; doi:10.15252/embr.201540467)
Supplement: Supplementary file 2 [file embr0016-1177-sd2.pdf]

Expanded View Figures

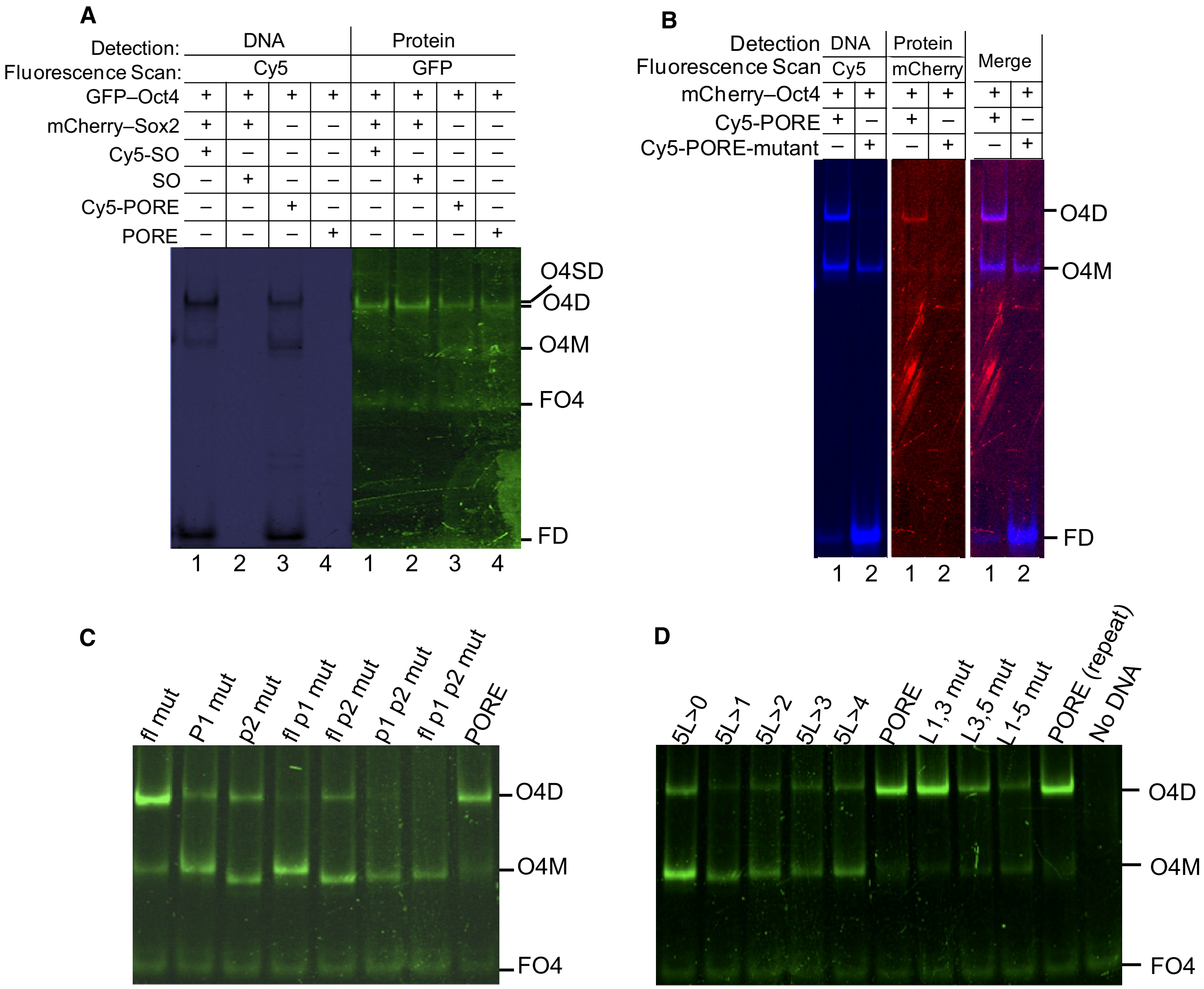

Figure EV1. Validating FP-EMSA.

A Homodimer formation was tested using DNA labelled with Cy5 or unlabelled as indicated. The presence of a Cy5 tag does not affect protein binding as dimerisation in lanes 2 and 4 is comparable with lanes 1 and 3.

B Homodimerisation is independent of the fluorescent protein tag. mCherry–Oct4 was tested instead of GFP-Oct4 with PORE-wt (lane 1) and PORE-mt (lane 2).

C Mutations inside the PORE motif affect homodimer formation, whereas mutations outside the PORE motif do not. The wild-type PORE is indicated; fl, P1 and P2 indicate oligonucleotides with mutations in the flanking sequence, palindromic repeat 1 and repeat 2, respectively.

D Effects of mutations of base pairs in the non-palindromic linker of the PORE motif (AAATG). 5L > 4-0 indicates sequential reduction in the size of the linker; other point mutation oligonucleotides are indicated.

Data information: O4D: GFP-Oct4 homodimer complex, O4SD: GFP-Oct4 and mCherry-Sox2 heterodimer complex, O4M: GFP-Oct4 monomer complex, FO4: free GFP-Oct4, and FD: free DNA. All oligonucleotide sequences are in Appendix Table S1. $n = 3$.
Source data are available online for this figure.

 © 2015 The Authors

A

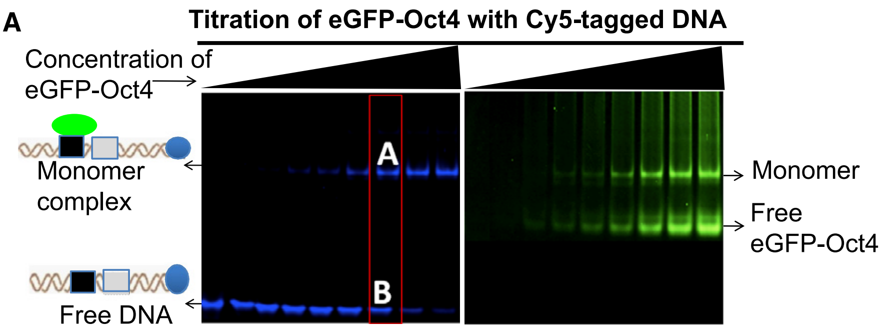

$$\text{Bound fraction} = \frac{\text{total complexes}}{\text{free DNA} + \text{total complexes}} = \frac{A}{A+B}$$

The apparent dissociation constant, aK_d, can be experimentally determined by ploting the bound fraction with respect to the concentration of the fusion protein.

B Sigmoidal fitting equation

$$Y(x) = Y_0 + \frac{Y_{max}}{1 + e^{\left(\frac{X_{half} - X}{R}\right)}}$$

Where Y(x) represents X (concentration of total protein) dependent bound fraction; Y0 is the base value; Ymax represents maximum bound fraction; Xhalf represents the concentration where the titration will reach to 50% of bound fraction; and R represents the rise rate. Smaller rate causes a faster rise.

Figure EV2. Quantification of aK_d by FP-EMSA.

A Blue panel: DNA; green panel: protein detection. For quantification purpose, only the blue panel needs to be taken into consideration. The bound fraction was calculated according to the equation provided.

B Sigmoid fitting function used to fit the bound fraction versus total protein concentration plot.

Source data are available online for this figure.

© 2015 The Authors

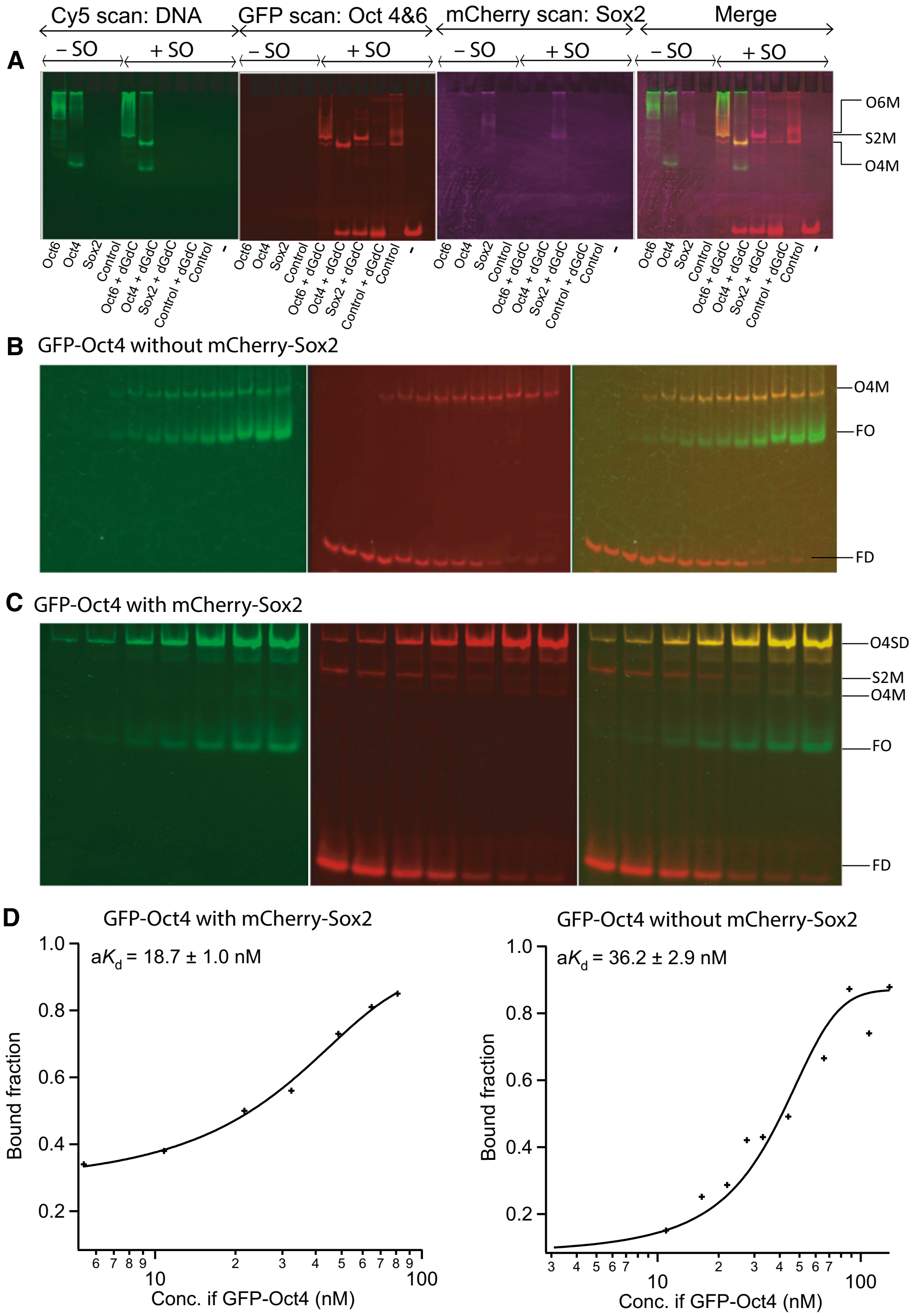

Figure EV3. Control experiments for the titration of Oct6 in the presence and absence of Sox2.

A Confirmative monomeric band was observed by studying proteins in the absence (−SO) or the presence (+SO) of the *Nanog* Sox/Oct sequence. Control is lysate from untransfected cells. dGdC is included as a non-specific competitor.

B, C FP-EMSA analysis of titrations of GFP-Oct4, binding to the *Nanog* Sox/Oct oligonucleotide (10 nM) in the absence (B) or the presence (C) of 132 nM mCherry-Sox2.

D Bound fractions observed from titrations shown in (B, C) were plotted against the respective concentration of Oct4 for quantifying the aK_d of those titrations.

Data information: O4SD: GFP-Oct4-mCherry-Sox2 dimer complex, O6M: GFP-Oct6 monomer complex, S2M: mCherry-Sox2 monomer complex, O4M: GFP-Oct4 monomer complex, FO: free GFP-Oct4, FD: free DNA. $n = 2$.
Source data are available online for this figure.

 © 2015 The Authors

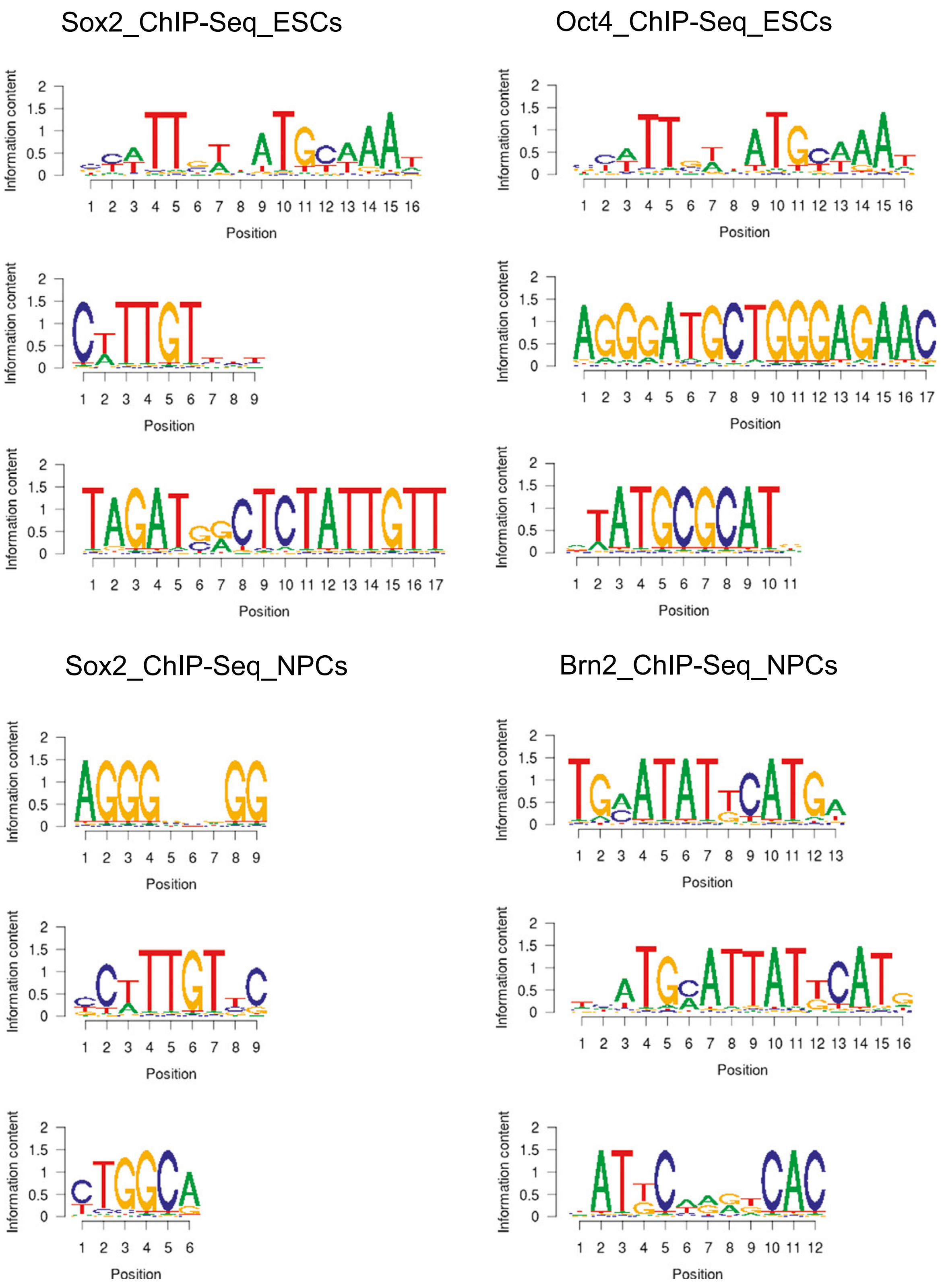

Figure EV4. *De novo* motif discovery from the published ChIP-Seq data (Lodato *et al*, 2013).
The top *de novo* sequence motifs (based on enrichment) detected by *XXmotif* (see Materials and Methods) in ChIP-Seq data for Oct4 in ESCs, Sox2 in ESCs (upper panel), Sox2 in NPCs and Brn2 in NPCs (lower panel). The top 3 enriched motifs for each are shown by the position frequency matrices visualised by WebLogo.
