# Supplementary material for: Selective influence of Sox2 on POU transcription factor binding in embryonic and neural stem cells
Source: EMBO Rep. 2015 Sep 2;16(9):1177–91. doi: 10.15252/embr.201540467 (PMC4576985; doi:10.15252/embr.201540467)
Supplement: Supplementary file 4 [file embr0016-1177-sd4.pdf]

EMBO
reports

Manuscript EMBOR-2015-40467

Selective influence of Sox2 on POU transcription factor binding in embryonic and neural stem cells

Tapan Kumar Mistri, Arun George Devasia, Lee Thean Chu, Wei Ping Ng, Florian Halbritter, Douglas Colby, Ben Martynoga, Simon R. Tomlinson, Ian Chambers, Paul Robson, Thorsten Wohland

Corresponding authors: Thorsten Wohland, Ian Chambers, Paul Robson

Review timeline:

Submission date:	30 March 2015
Editorial Decision:	27 April 2015
Revision received:	12 June 2015
Editorial Decision:	29 June 2015
Revision received:	02 July 2015
Accepted:	06 July 2015

Editor: Esther Schnapp

Transaction Report:

(Note: With the exception of the correction of typographical or spelling errors that could be a source of ambiguity, letters and reports are not edited. The original formatting of letters and referee reports may not be reflected in this compilation.)

1st Editorial Decision 27 April 2015

Thank you for the transfer of your research manuscript to EMBO reports. I apologize for the delay in getting back to you, I was not in the office last week. We have received last week all referee reports that are included below.

As you will see, all referees acknowledge that the study is well done and presented. Given that they only have a few suggestions for how the work can be improved, I would like you to address all of them.

Given these constructive comments, we would like to invite you to revise your manuscript with the understanding that the referee concerns (as mentioned above and in their reports) must be fully addressed and their suggestions taken on board. Acceptance of the manuscript will depend on a positive outcome of a second round of review. It is EMBO reports policy to allow a single round of revision only and acceptance or rejection of the manuscript will therefore depend on the completeness of your responses included in the next, final version of the manuscript.

Regarding data quantification, can you please specify the number "n" for how many experiments were performed, along with the bars and error bars (e.g. SEM, SD) and the test used to calculate p-values in the respective figure legends? This information is currently incomplete and must be provided in the figure legends.

I look forward to seeing a revised version of your manuscript when it is ready. Please let me know if you have questions or comments regarding the revision.

REFEREE REPORTS:

Referee #1:

Mistri et al. provide a wonderful and technically superb manuscript, in which they systematically study Sox2-POU factor interactions in NSCs and ESCs. By combining Chip-Seq, Fluorescent-EMSA (developed and applied in this study) and exacting mutants, they established a number of important observations:
1) in ESCs OCt4/Sox2 synergistically bind Oct4/Sox motif as heterodimers (as previously shown).
2) Remarkably, in NSCs POU factors (Oct6 or Brn1) does no form hetero-dimers with Sox2, but rather bind as homo-dimers on MORE cis motif, rather than Oct4/Sox2 motif. This binding is not influenced or deemed synergistic with Sox2.

The manuscript is excellently written with a great introduction explaining the current state of the field. The conclusions are well supported by the data. The discussions are insightful. Method section is very detailed, and references are adequate throughout the manuscript. re-analyzing Lodato et al. data and conclusions is important, fair and relevant.

I recommend publishing this superb work as is.

Referee #2:

In this manuscript Wohland and colleagues show how the transcription factor (TF) Sox2 and other Oct homologues interact in embryonic stem (ES) cells and neural stem (NS) cells.

They use biochemical approaches to test the hypothesis that Sox2 changes partners in different cell types.

Their experiments convincingly show that in NS cells Sox2 and class III POU TFs do not act cooperatively. Instead, POU TFs interact with MORE elements.

This study extends our knowledge of the biochemical interactions controlling pluripotent and multipotent stem cells. The experiments are carefully performed and the results are well presented. The findings also contribute to understanding how TFs change between homo- and heterodimerization states.

Their characterization of different Sox-Oct interactions occurring in different cell typed may also guide future improvements in reprogramming strategies.

I suggest that the authors address two important technical issues:

1) aKd determination: It is unclear if the bound fraction is plotted against free protein concentration or total protein concentration. The authors need to describe the model used, perhaps adding the relevant equations in the main text and explaining if any approximation was taken.

2) The authors state that they are capable of identifying free and bound Cy5 DNA oligos in the fluorescence correlation spectroscopy (FCS) curves by fitting their ACFs with a two-population model. Temporal separation of both populations is critical to the identification of independent populations. The authors should show the ACF curves with clearly distinguishable components.

Finally, although the manuscript is clearly written, the entire text could be shortened. A few concepts are repeated in introduction, results and discussion.

Also, please choose between ESC / NCS or ES cells / NS cells nomenclature to avoid

inconsistencies.

Referee #3:

In this manuscript, the authors reported the differential characters of class III and class V POU transcription factors for interaction with Sox2. They applied FP-EMSA to examine the interaction of Oct6 (Class III) and Oct4 (Class V) for their cooperative binding to the target sequence with Sox2 and found that Oct4 preferentially form heterodimer with Sox2 whereas Oct6 tend to form homodimer. Then they analyzed the published ChIP-seq data and confirmed that Oct4 mainly bind to Oct-Sox motif in embryonic stem (ES) cells and Oct6 and other class III POU factors often occupy palindromic Oct motif. The analyses performed in well-organized way and the results sounds quite solid, so I basically agree with the publication of this manuscript. However, there is a sole concern about the application of fusion proteins in FP-EMSA assay. Are these fusion proteins functionally equivalent to wild type proteins? This is important to interpret the result obtained by FP-EMSA, so I would like to ask the authors to confirm their function in physiological context.

1st Revision - authors' response — 12 June 2015

Reply to the Reviewers#

Referee #1

Comment: Mistri et al. provide a wonderful and technically superb manuscript, in which they systematically study Sox2-POU factor interactions in NSCs and ESCs. By combining Chip-Seq, Fluorescent-EMSA (developed and applied in this study) and exacting mutants, they established a number of important observations:

1) in ESCs OCt4/Sox2 synergistically bind Oct4/Sox motif as heterodimers (as previously shown).

2) Remarkably, in NSCs POU factors (Oct6 or Brn1) do no form hetero-dimers with Sox2, but rather bind as homo-dimers on MORE cis motif, rather than Oct4/Sox2 motif. This binding is not influenced or deemed synergistic with Sox2.

The manuscript is excellently written with a great introduction explaining the current state of the field. The conclusions are well supported by the data. The discussions are insightful. Method section is very detailed, and references are adequate throughout the manuscript. re-analyzing Lodato et al. data and conclusions is important, fair and relevant.

I recommend publishing this superb work as is.

Answer: *Thank you for the appreciation.*

Referee #2:

© European Molecular Biology Organization

Comments: In this manuscript Wohland and colleagues show how the transcription factor (TF) Sox2 and other Oct homologues interact in embryonic stem (ES) cells and neural stem (NS) cells.

They use biochemical approaches to test the hypothesis that Sox2 changes partners in different cell types.

Their experiments convincingly show that in NS cells Sox2 and class III POU TFs do not act cooperatively. Instead, POU TFs interact with MORE elements.

This study extends our knowledge of the biochemical interactions controlling pluripotent and multipotent stem cells. The experiments are carefully performed and the results are well presented. The findings also contribute to understanding how TFs change between homo- and heterodimerization states.

Their characterization of different Sox-Oct interactions occurring in different cell typed may also guide future improvements in reprogramming strategies.

I suggest that the authors address two important technical issues:

1) aK_d determination: It is unclear if the bound fraction is plotted against free protein concentration or total protein concentration. The authors need to describe the model used, perhaps adding the relevant equations in the main text and explaining if any approximation was taken.

Answer: *Sorry for the confusion. We would like to draw the reviewers' attention to Extended View Figure 2 (Previously Fig S2). This Figure has been updated and a further explanatory section (B) added for clarity. The bound fraction is plotted against total protein concentration which is measured by FCS assay. We used standard sigmoidal fitting module to determine the protein concentration required for 50% bound fraction of the protein-DNA complex.*

2) The authors state that they are capable of identifying free and bound Cy5 DNA oligos in the fluorescence correlation spectroscopy (FCS) curves by fitting their ACFs with a two-population model. Temporal separation of both populations is critical to the identification of independent populations. The authors should show the ACF curves with clearly distinguishable components.

© European Molecular Biology Organization

Answer: *This is an important point. We have now provided a supplementary figure (**Fig. S2**) that provides the FCS curves, their fits and the fit residuals for one- and two-component models. From the residuals it can be seen that for the case of Cy5-DNA alone in solution, the two-component model does not significantly improve on the fit residuals which are evenly distributed around zero. In the case of Cy5-DNA in solution with either low (40nM) or high (150nM) concentration the residuals for the one-component fit show characteristic undulations around the zero line which are characteristic for non-proper fits. In both cases, the two-component model fits significantly better and reduces these undulations, restoring an almost equal random distribution of residuals around zero.*

3) Finally, although the manuscript is clearly written, the entire text could be shortened. A few concepts are repeated in introduction, results and discussion.

Answer: *We have re-written the paper to remove these redundancies. We have done this without compromising the clarity of writing or the "great introduction" and "insightful" discussion commented on by reviewer 1.*

4) Also, please choose between ESC / NCS or ES cells / NS cells nomenclature to avoid inconsistencies.

Answer: *We now refer to cells as ESCs or NSCs throughout the manuscript.*

Referee #3:

Comment: In this manuscript, the authors reported the differential characters of class III and class V POU transcription factors for interaction with Sox2. They applied FP-EMSA to examine the interaction of Oct6 (Class III) and Oct4 (Class V) for their cooperative binding to the target sequence with Sox2 and found that Oct4 preferentially form heterodimer with Sox2 whereas Oct6 tend to form homodimer. Then they analyzed the published ChIP-seq data and confirmed that Oct4 mainly bind to Oct-Sox motif in embryonic stem (ES) cells and Oct6 and other class III POU factors often occupy palindromic Oct motif. The analyses performed in well-organized way and the results sounds quite solid, so I basically agree with the publication of this manuscript. However, there is a sole concern about the application of fusion proteins in FP-EMSA assay. Are these fusion proteins functionally equivalent to wild type proteins? This is important to interpret the result obtained by FP-EMSA, so I would like to ask the authors to confirm their function in physiological context.

Answer: *We thank the reviewer for pointing out this important omission. We have tested the function of GFP-Oct4 and mCherry-Sox2 by complementation assays in ESCs in direct comparison to wild-type Oct4 and Sox2 respectively. For these experiments, we used the*

ESC lines ZHBTc4 [1] and SCKO [2]. ZHBTc4 cells have both Oct4 alleles deleted and is sustained in a pluripotent state by a doxycycline-suppressible Oct4 transgene. Application of Doxycycline to these cells silences the Oct4 transgene and causes differentiation to a trophectodermal state. SCKO cells have one Sox2 allele deleted and the other flanked by loxP sites. These cells also express a constitutive Cre-ERT2 fusion protein which is held in an inactive state in the absence of Tamoxifen. Application of Tamoxifen stimulates the Cre activity of the Cre-ERT2 fusion, deletes the remaining Sox2 allele and drives trophectodermal differentiation. Transfection of ZHBTc4 cells with GFP-Oct4 and Oct4 resulted in the same number of undifferentiated ESCs, whereas transfection of an empty vector resulted in loss of self-renewal. Likewise, transfection of SCKO cells with mCherry-Sox2 or Sox2 resulted in comparable numbers of undifferentiated ESCs, whereas empty vector transfections resulted in differentiation. This analysis of biological activity of the fusion constructs is now stated in the Results and the data presented in ***Fig. S1.***

For Oct6, there is not an established equivalent physiological assay. However, Zhu et al have shown that overexpression of Oct6 in ES cells undergoing 8 days of "unbiased" differentiation in embryoid body suspension without LIF or feeder cells, results in increased expression of the neural lineage marker Nestin as well as Zic1, a gene identified by us as a target of POU-TFs in NSCs [3].

Therefore, to test the function of GFP-Oct6, ESCs were transfected with a GFP-Oct6 vector or control vector. After 6 days continued culture in ES cell conditions, expression of the neural marker Nestin and the Oct6 target genes Zic1 and Kdm2b (which we also identify by ChIP-Seq as an NSC POU-TF target) are elevated. As our cells are grown under non-differentiating conditions for a shorter time than used by Zhu et al, our conditions represent a greater barrier to NSC gene expression than the conditions used by Zhu et al [3]. This validation of the biological activity of the GFP-Oct6 fusion is now stated in the Results and the data presented in ***Fig. S1E****.*

We consider that these cell based assays meet the reviewers request for physiological demonstration of the functionality of the various FP-TF fusions that we have used.

REFERENCES

1. Niwa H, Miyazaki J-i, Smith AG (2000) Quantitative expression of Oct-3/4 defines differentiation, dedifferentiation or self-renewal of ES cells. *Nature genetics* **24:** 372-376

2. Gagliardi A, Mullin NP, Ying Tan Z, Colby D, Kousa AI, Halbritter F, Weiss JT, Felker A, Bezstarosti K, Favaro R (2013) A direct physical interaction between Nanog and Sox2 regulates embryonic stem cell selfâ€ renewal. *The EMBO journal* **32:** 2231-2247

3. Zhu Q, Song L, Peng G, Sun N, Chen J, Zhang T, Sheng N, Tang W, Qian C, Qiao Y (2014) The transcription factor Pou3f1 promotes neural fate commitment via activation of neural lineage genes and inhibition of external signaling pathways. *ELife* **3:** e02224

2nd Editorial Decision 29 June 2015

Thank you for the submission of your revised manuscript to EMBO reports. As you will see, both referees support publication of the revised study, and I am happy to tell you that we can therefore in principle accept it.

However, going through the files I noticed that the legends for Figure 3C and EV3D state n=2. If n<3 no statistics can be calculated and no error bars shown. Please either repeat the experiments at least one more time or remove the error bars and show the individual data points along with their mean instead.
Not all questions, especially regarding statistics (points 1-5), have further been answered in the author checklist. Please note that page numbers must be listed for all applicable points in the checklist in order to ensure that the information is present in the manuscript file. Please answer all questions under "B statistics" and list the page numbers where this information can be found for B-G.

Please define the scale bar in SF1A.

In our supplementary information (now called Appendix) no materials and methods are allowed. Please move all materials and methods to the main manuscript file.

I am looking forward to receiving the final manuscript files.

REFEREE REPORTS:

Referee #2:

The authors have addressed all my questions satisfactorily with new explanations and additional data.

I now fully support publication of this manuscript.

Referee #3:

In this revised manuscript, the authors supplied proper answers to the reviewers' comments. Now I agree with the publication of present version of manuscript as it is.

2nd Revision - authors' response 02 July 2015

Reply to the Editor

Dear Editor,
Thank you for your email stating acceptance of our manuscript in principle. Below we provide point by point responses to the remaining issues you have raised:

1. However, going through the files I noticed that the legends for Figure 3C and EV3D state n=2. If n<3 no statistics can be calculated and no error bars shown. Please either repeat the experiments at

least one more time or remove the error bars and show the individual data points along with their mean instead.

Answer: We chose to the 2nd option and acted as you suggested by plotting the graphs with the mean value of individual data points without the error bars.

2. Not all questions, especially regarding statistics (points 1-5), have further been answered in the author checklist. Please note that page numbers must be listed for all applicable points in the checklist in order to ensure that the information is present in the manuscript file. Please answer all questions under "B statistics" and list the page numbers where this information can be found for B-G.

Answer: We believe we have answered all relevant questions in the author checklist.

3. Please define the scale bar in SF1A.

Answer: The scale bar is 10 µM. We corrected this in the revised version of SF1A.

4. In our supplementary information (now called Appendix) no materials and methods are allowed. Please move all materials and methods to the main manuscript file.

Answer: We have amended this. Now all the materials and methods are in the main text.

3rd Editorial Decision 06 July 2015

I am very pleased to accept your manuscript for publication in the next available issue of EMBO reports.

Thank you for your contribution to EMBO reports and congratulations on a successful publication. Please consider us again in the future for your most exciting work.
