# Supplementary figures and images for: Selective influence of Sox2 on POU transcription factor binding in embryonic and neural stem cells
Source: EMBO Rep. 2015 Sep 2;16(9):1177–91. doi: 10.15252/embr.201540467 (PMC4576985; doi:10.15252/embr.201540467)

Mistri et al.

## Figure 1

Fig. 1A, 1B

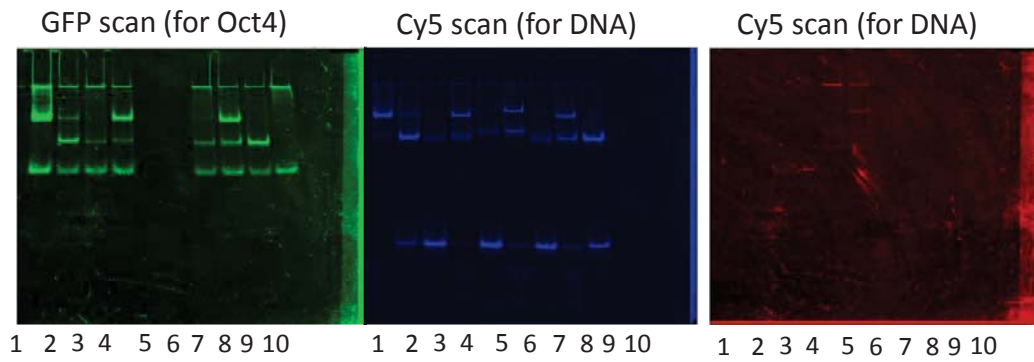

Lane 1-3: Fig1A  
Lane 4: GFP-Oct4+MORE  
Lane 5-6: Fig. EV1B  
Lane 7-10: Fig. 1A

Fig. 1C

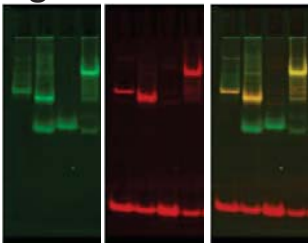

Fig. 1D

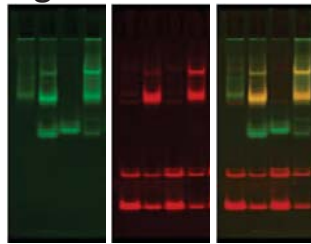

Supplement: Supplementary file 5 [file embr0016-1177-sd5.pdf]

Fig. 6A

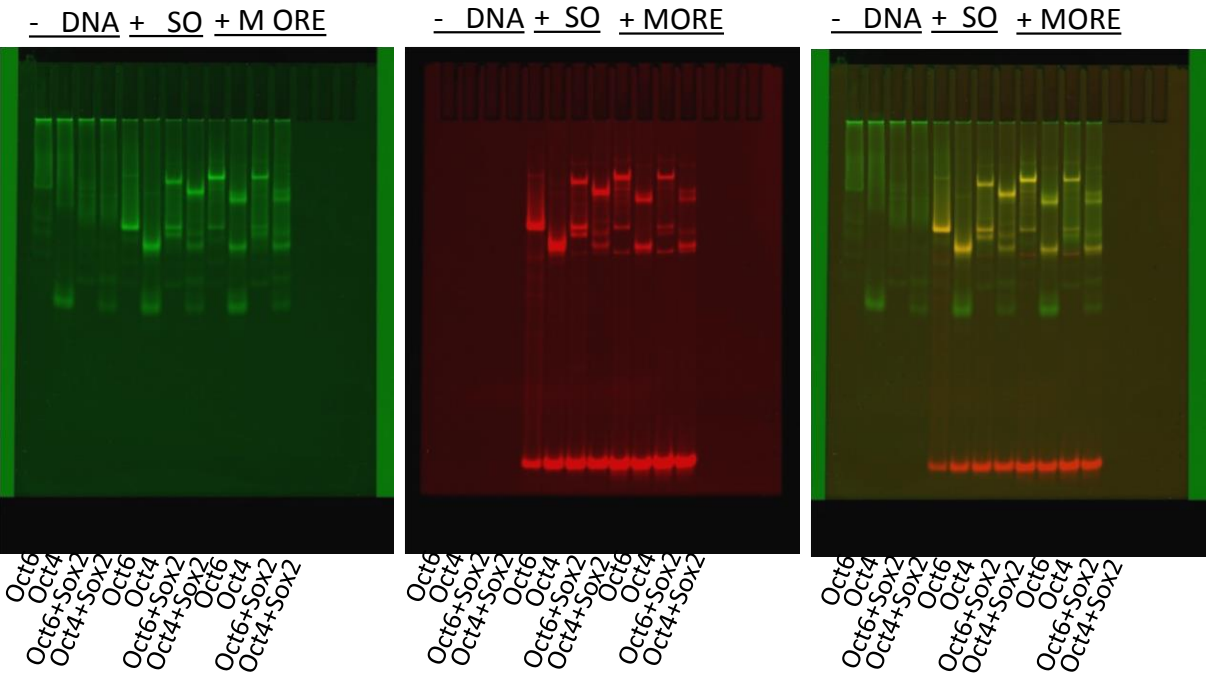

PORE panel of Fig. 6A and Fig. 6B

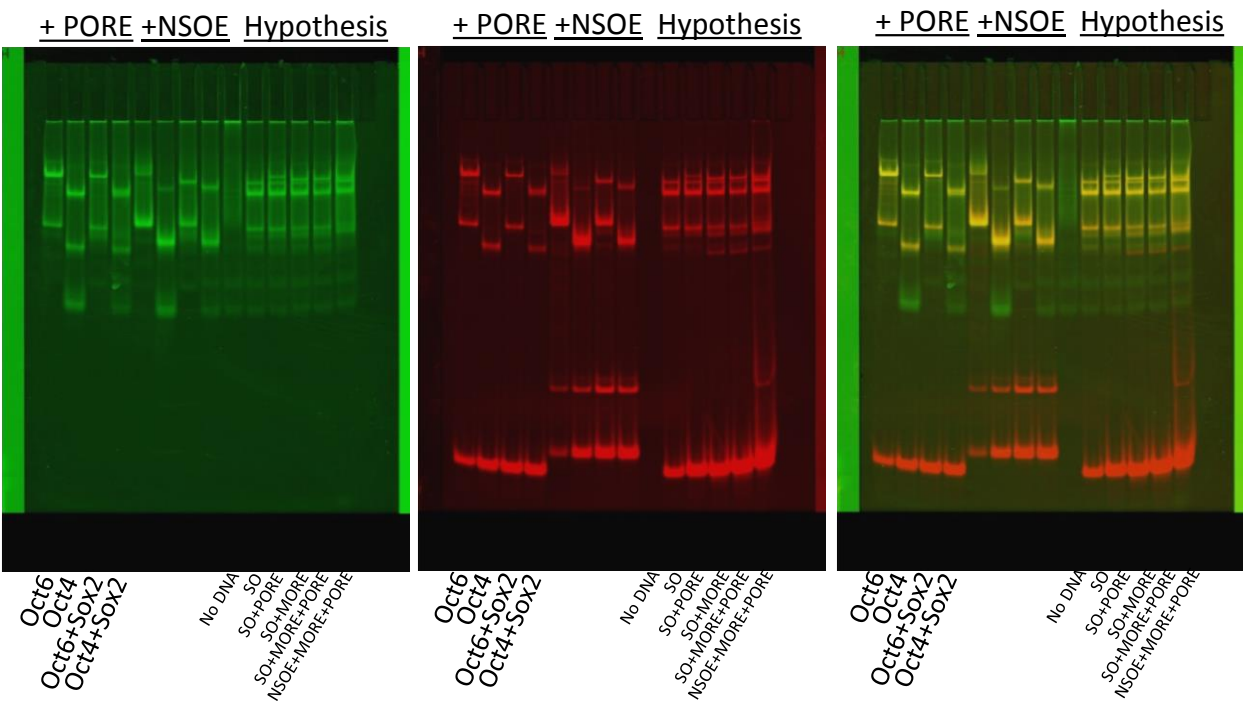

Supplement: Supplementary file 9 [file embr0016-1177-sd9.pdf]

# Mistri et al.

## Appendix Figure S1

**Fig. S1A**

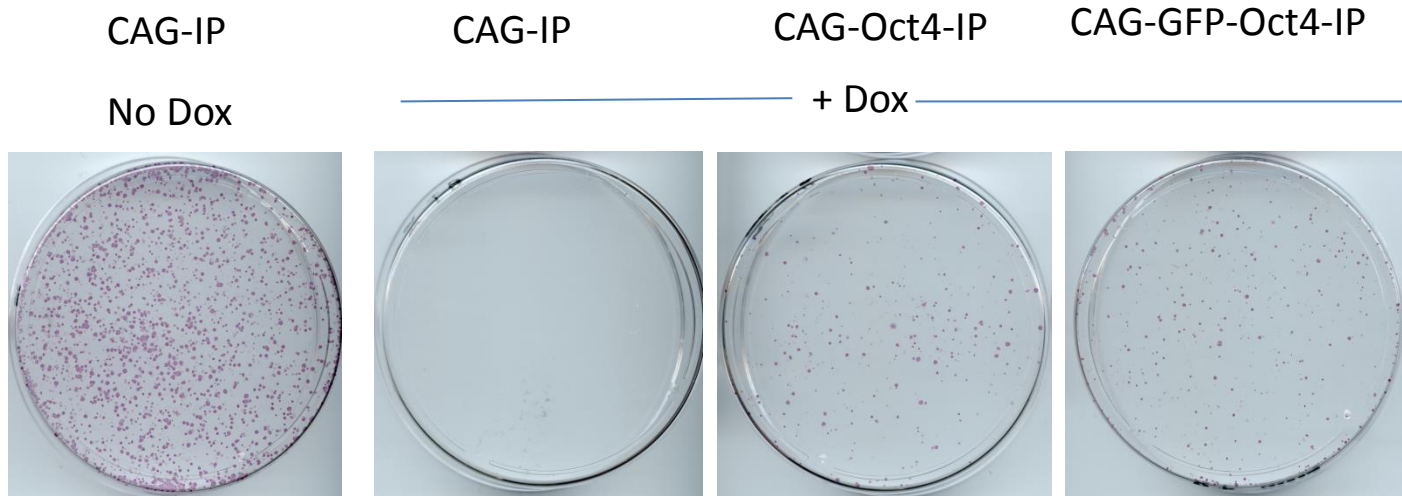

**Fig. S1C**

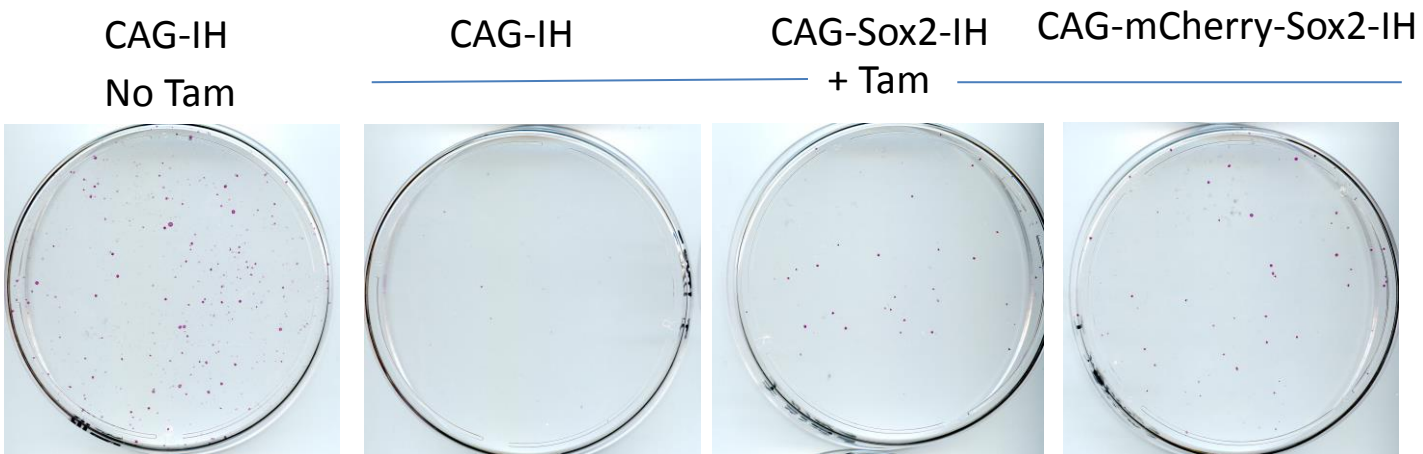

Supplement: Supplementary file 10 [file embr0016-1177-sd10.zip › Source Data for Expanded View and Appendix/Source Data for Appendix/Source_Data_Appendix_Figure_S1.pdf]

Mistri et al.  
Figure EV1

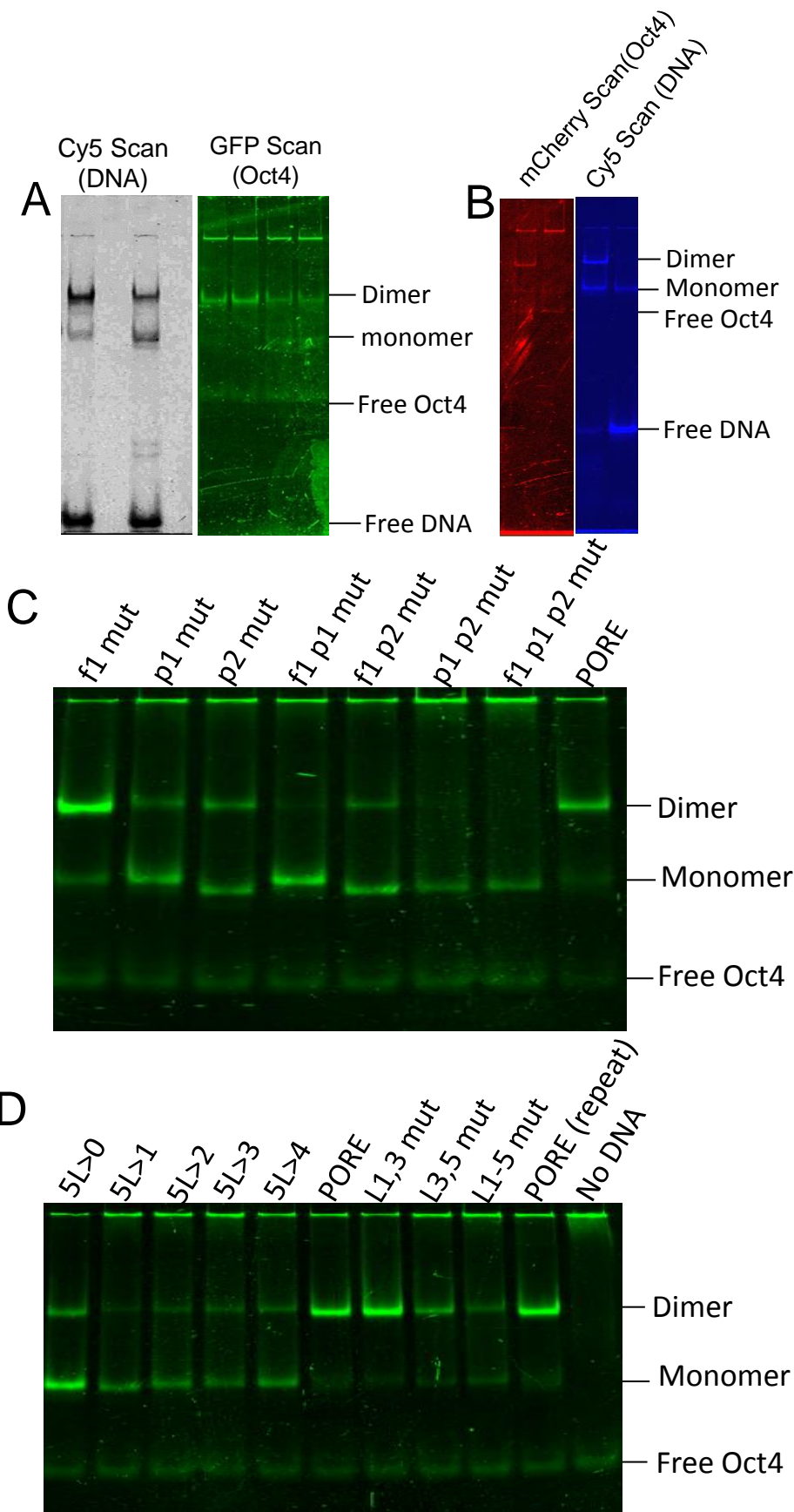

Supplement: Supplementary file 10 [file embr0016-1177-sd10.zip › Source Data for Expanded View and Appendix/Source Data for Expanded View/Source_Data_Figure_EV1.pdf]
