# Supplementary material for: Selective influence of Sox2 on POU transcription factor binding in embryonic and neural stem cells
Source: EMBO Rep. 2015 Sep 2;16(9):1177–91. doi: 10.15252/embr.201540467 (PMC4576985; doi:10.15252/embr.201540467)

Mistri et al.
Figure 3

Fig. 3A

Titration of GFP-Oct6 in the absence of mCherry-Sox2

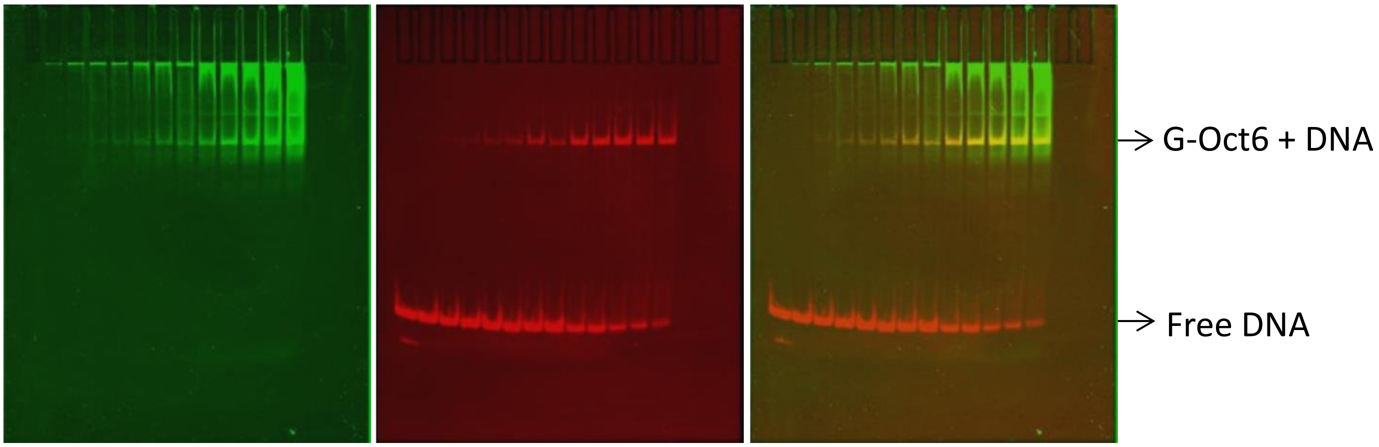

Fig. 3B

Titration of GFP-Oct6 in the presence of mCherry-Sox2

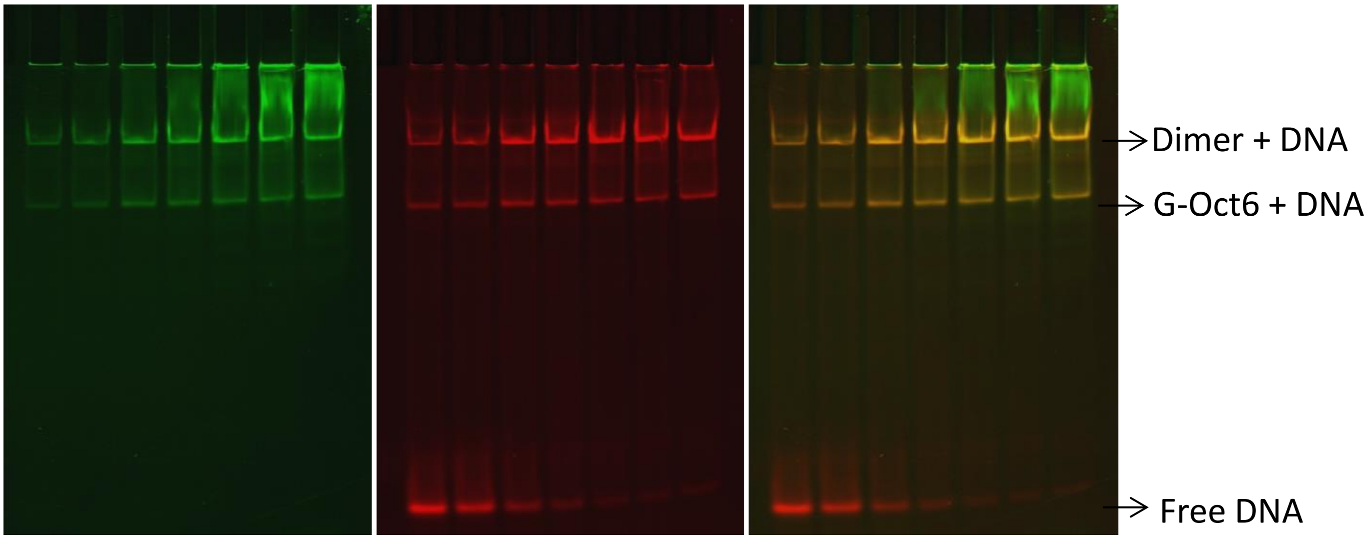

Supplement: Supplementary file 7 [file embr0016-1177-sd7.pdf]
