# Supplementary material for: Selective influence of Sox2 on POU transcription factor binding in embryonic and neural stem cells
Source: EMBO Rep. 2015 Sep 2;16(9):1177–91. doi: 10.15252/embr.201540467 (PMC4576985; doi:10.15252/embr.201540467)

Mistri et al.
Figure 4

Fig 4A

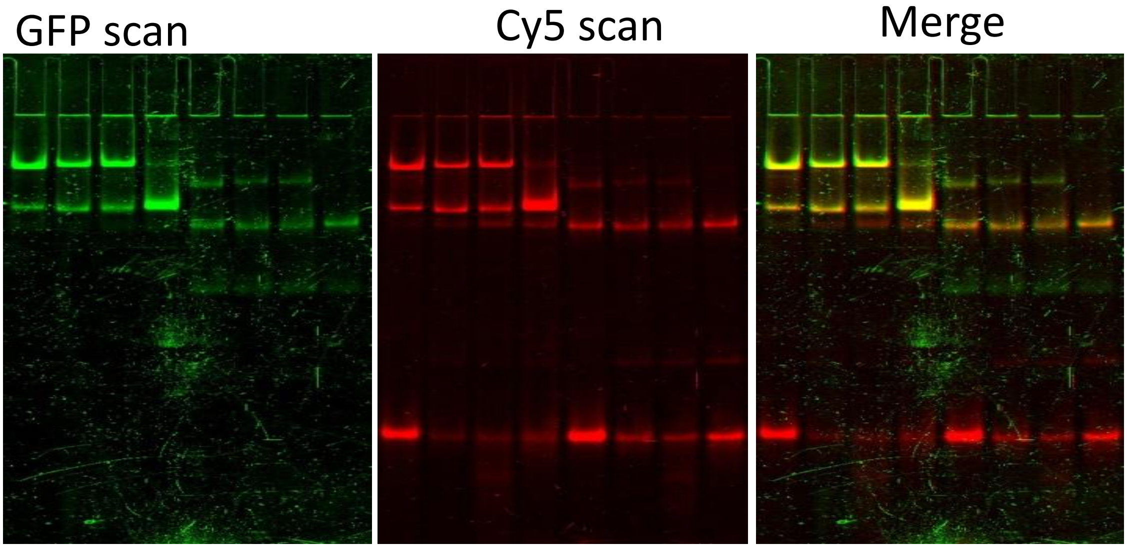

Fig 4B

Standard EMSA with Cy5 based gel scanning (DNA detection)

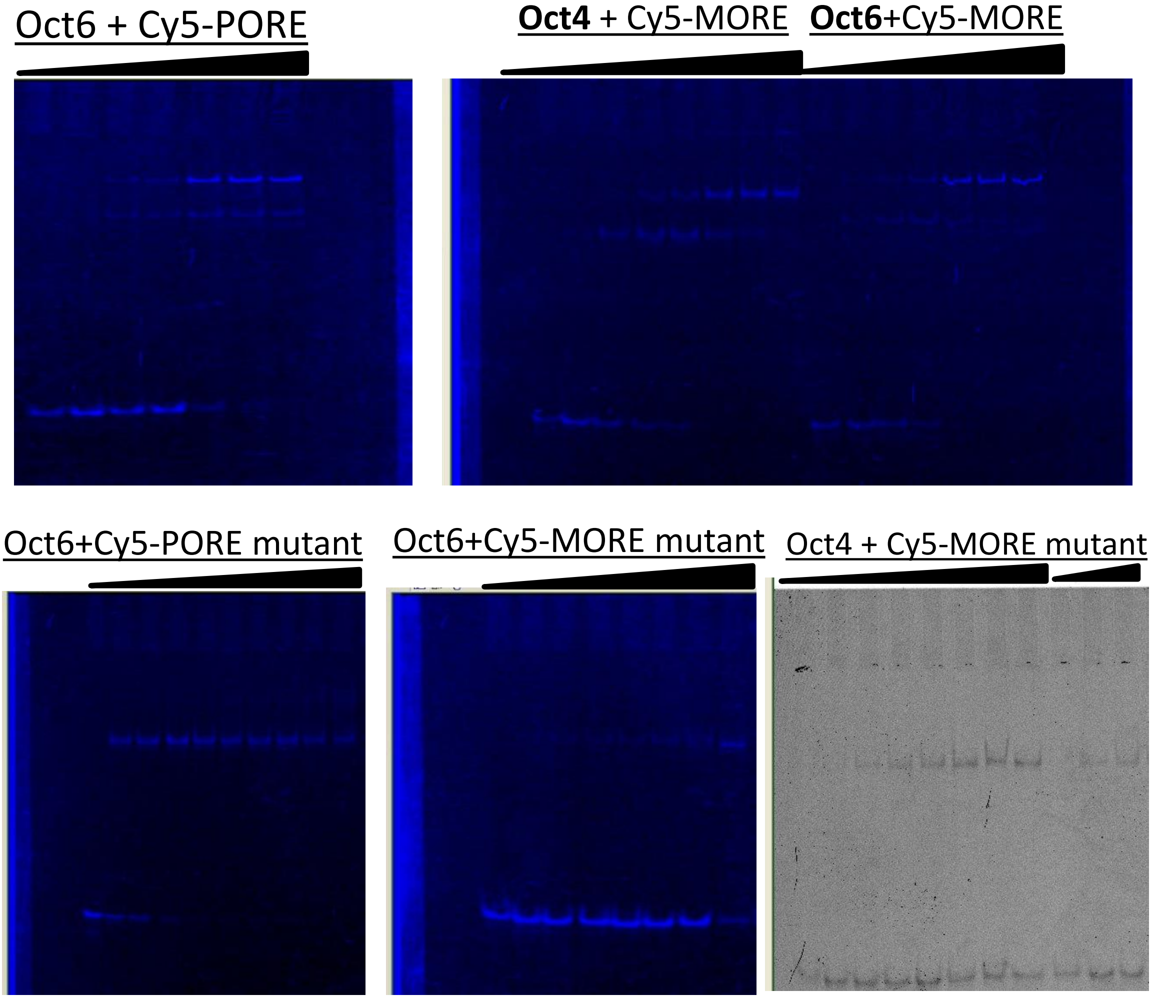

Supplement: Supplementary file 8 [file embr0016-1177-sd8.pdf]
