# Supplementary material for: Selective influence of Sox2 on POU transcription factor binding in embryonic and neural stem cells
Source: EMBO Rep. 2015 Sep 2;16(9):1177–91. doi: 10.15252/embr.201540467 (PMC4576985; doi:10.15252/embr.201540467)

Mistri et al.
Figure EV2

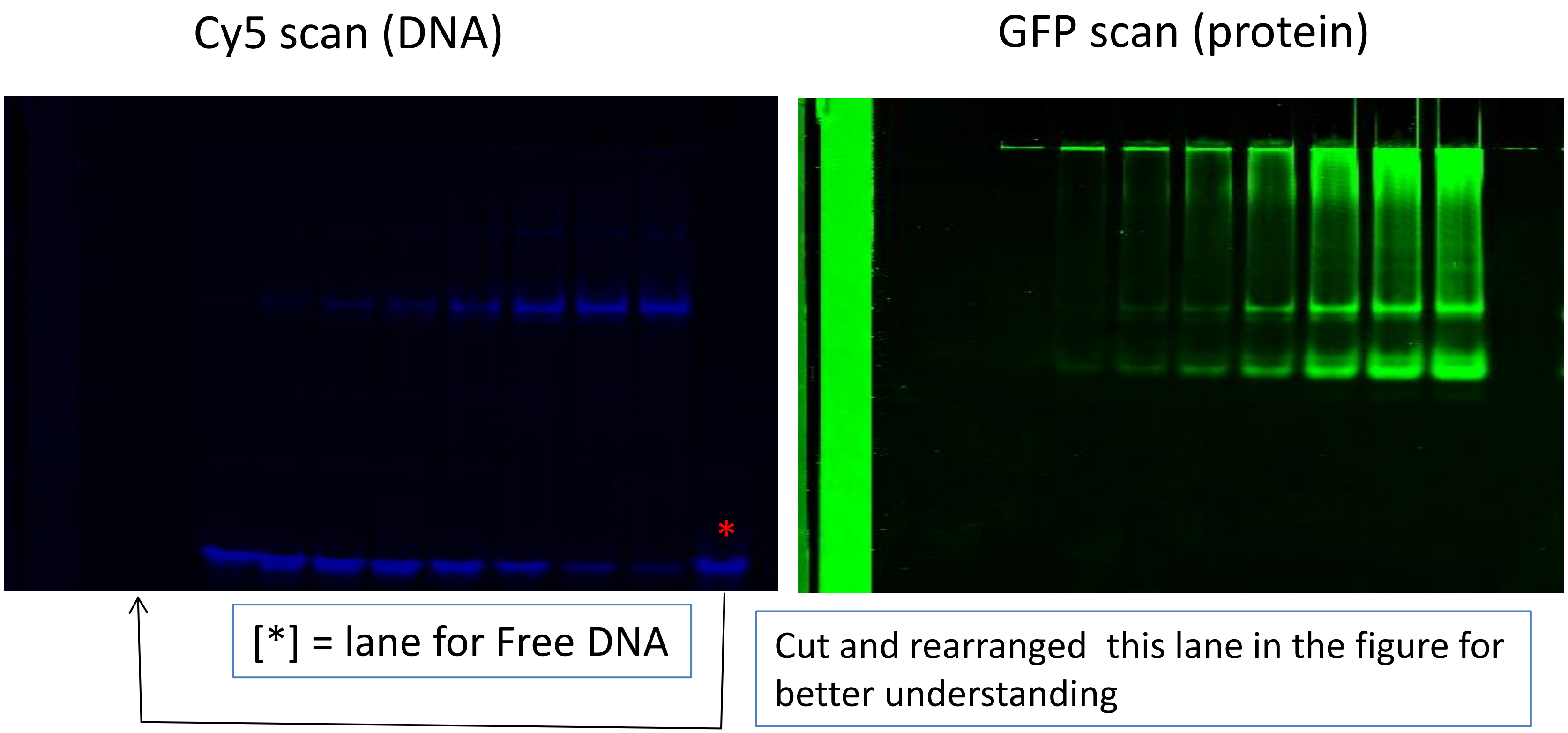

Supplement: Supplementary file 10 [file embr0016-1177-sd10.zip › Source Data for Expanded View and Appendix/Source Data for Expanded View/Source_Data_Figure_EV2.pdf]
